# Blocking RAN translation without altering repeat RNAs rescues *C9ORF72*-related ALS and FTD phenotypes

**DOI:** 10.1126/science.adv2600

**Published:** 2026-02-05

**Authors:** Xin Jiang, Laure Schaeffer, Divya Patni, Tommaso Russo, Chao-Zong Lee, Corey Aguilar, Christine Marques, Karen Jansen-West, Marian Hruska-Plochan, Ananya Ray-Soni, Su Min Lim, Aaron Held, Mei Yue, Paula Castellanos Otero, Sandeep Aryal, Hortense D. A. M. Beaussant, Himanish Basu, Hiro Takakuwa, Lillian M. Daughrity, Nandini Ramesh, Paulo Da Costa, Ana Rita A. A. Quadros, Matthew Nolan, Charles Jourdan F. Reyes, Hayden Wheeler, Laura C. Moran, Grant Griesman, Benjamin Wymann, Bianca A. Trombetta, Emma Sofia Lopez-De-Silanes, Michael Canori, Gopinath Krishnan, Yasmim Vieira Souza Da Silva, Gilbert Eriani, Mark W. Albers, Steven E. Arnold, Yuyu Song, Ankur Jain, Isaac M. Chiu, Yong-Jie Zhang, Fen-Biao Gao, Brian J. Wainger, Magdalini Polymenidou, Leonard Petrucelli, Franck Martin, Clotilde Lagier-Tourenne

**Affiliations:** 1 Department of Neurology, Massachusetts General Hospital, Harvard Medical School, Boston, MA, USA; 2 Broad Institute of Harvard University and MIT, Cambridge, MA, USA; 3 Université de Strasbourg, Institut de Biologie Moléculaire et Cellulaire, Architecture et Réactivité de l’ARN, CNRS UPR9002, Strasbourg, France; 4 Neurology Unit and Experimental Neuropathology Unit, Institute of Experimental Neurology, Division of Neuroscience, IRCCS San Raffaele Scientific Institute, Milan, Italy; 5 Department of Neuroscience, Mayo Clinic, Jacksonville, FL, USA; 6 Department of Quantitative Biomedicine, University of Zurich, Zurich, Switzerland; 7 Department of Immunology, Harvard Medical School, Boston, MA, USA; 8 Whitehead Institute for Biomedical Research, Cambridge, MA, USA; 9 Frontotemporal Dementia Research Center, RNA Therapeutics Institute, University of Massachusetts Chan Medical School, Worcester, MA, USA; 10 Marine Biological Laboratory, Woods Hole, MA, USA; 11 Department of Biology, Massachusetts Institute of Technology, Cambridge, MA, USA

## Abstract

GGGGCC (G_4_C_2_) repeat expansion in *C9ORF72* is the most common genetic cause of amyotrophic lateral sclerosis (ALS) and frontotemporal dementia (FTD). Toxicity is thought to result from the accumulation of either repeat RNAs and/or dipeptide repeat proteins (DPRs) translated from repeat-containing transcripts through repeat-associated non-AUG (RAN) translation. To disentangle RNA from DPR toxicity, we mutated a CUG codon predominantly used to initiate DPR translation from all three reading frames. This mutation disrupted DPR synthesis while preserving the expression of repeat-containing RNAs. Despite the accumulation of RNA foci, behavioral deficits and pathological abnormalities, including p-TDP-43 inclusions, STING activation, motor neuron loss, neuroinflammation, and increased plasma neurofilament concentration, were alleviated in *C9ORF72* mice. base editing of the CUG codon also improved molecular phenotypes and survival in patient induced pluripotent stem cell–derived neurons, which highlights the potential of therapeutically targeting DPR production rather than repeat RNAs.

Amyotrophic lateral sclerosis (ALS) and frontotemporal dementia (FTD) are two clinically distinct neurodegenerative diseases that exhibit genetic and pathological overlap (1). ALS primarily affects motor function due to the loss of upper and lower motor neurons (2). FTD results from neuronal loss in the frontal and temporal lobes, leading to language difficulties and changes in behavior and personality (3). ALS and FTD share neuropathological features and common genetic causes, including GGGGCC (G_4_C_2_) hexanucleotide repeat expansions within the first intron of the *C9ORF72* gene (4, 5).

Both reduced expression of the C9ORF72 protein and gain of toxic function from the repeat expansion have been proposed to contribute to disease (6–9), with loss of the C9ORF72 protein exacerbating gain of toxicity in mice (10). Toxic gain of function may arise either from the accumulation of RNA foci containing transcripts from sense and antisense strands (5, 11–13) or from the aberrant translation of these transcripts into five dipeptide repeat proteins (DPRs): poly-GA and poly-GR from the sense strand, poly-PA and poly-PR from the antisense strand, and poly-GP from both strands (6–8). RNA foci were proposed to sequester RNA binding proteins (RBPs) and induce RNA processing alterations (14–16), though there is no consensus as to which RBP(s) is or are functionally disrupted in *C9ORF72* disease (13, 17). By contrast, substantial evidence supports deleterious effects from the expression of DPRs, especially aggregation-prone poly-GR, poly-PR, and poly-GA that disrupt several cellular processes (8, 18). Unraveling the relative contribution of RNA-mediated toxicity versus DPR-mediated toxicity has been challenging because repeat-containing RNAs produce DPR proteins through repeat-associated non-AUG (RAN) translation, which precludes clear separation between both mechanisms (17). Evidence against RNA-mediated toxicity is supported by studies using *Drosophila* expressing G_4_C_2_ repeats interrupted with stop codons every 12 repeats to abolish the production of DPRs while preserving the formation of nuclear and cytoplasmic RNA foci (19, 20). Degeneration was absent in flies expressing the “RNA-only” constructs, which supports DPRs as major drivers of toxicity, although it cannot be excluded that interruptions within the repeat altered the structure of the RNAs and disrupted binding by RBPs (19, 20). Cytoplasmic transport of expanded RNAs is influenced by surrounding sequences and is crucial for the production of DPRs (21). Indeed, expanded G_4_C_2_ repeats flanked by human intronic sequences produced fewer DPR proteins than repeats in the context of polyA^+^ mRNA, and modest toxicity observed in this *Drosophila* model correlated with the amounts of DPR proteins but not the number of nuclear RNA foci, further supporting the notion that DPR proteins are the major source of toxicity (22, 23). Conversely, other studies have reported model organisms developing phenotypes despite undetectable DPRs (24, 25), which supports a role for RNA toxicity with the caveat that detection of DPRs may vary depending on the sensitivity of the biochemical assay used (8).

In this work, we disentangled RNA from protein toxicity by exploiting our previous discovery that DPRs from all three frames are predominantly generated from translation starting at the same CUG start codon located upstream of the G_4_C_2_ repeats in the poly-GA frame (26). The production of not only poly-GA but also poly-GP and poly-GR was abolished in vitro upon a single point mutation of the CUG codon into a nonfunctional CCG codon, providing evidence for a frameshifting mechanism in *C9ORF72* disease (26). Recognizing that the frameshifting allowed us to alleviate DPR production while conserving repeat-containing transcripts intact, we now show that editing a single base pair in the CUG translation initiation codon alleviates disease-associated phenotypes both in *C9ORF72* mice and *C9ORF72* patient induced pluripotent stem cell (iPSC)–derived neurons. Our findings support that DPRs, rather than repeat RNAs, primary drive toxicity in *C9ORF72*-related ALS and FTD (*C9ORF72*-ALS/FTD).

## Production of DPRs in all three frames initiates at a single CUG codon upstream of the *C9ORF72* G_4_C_2_ repeats

We and others have identified a near-cognate CUG start codon located 24 nucleotides upstream of the G_4_C_2_ repeats to be essential for the production of poly-GA (26–28). We have also demonstrated that mutation of the CUG codon into CCG disrupted the production of poly-GP (+1 frame) and poly-GR (+2 frame), providing initial evidence for a frameshifting mechanism in the production of DPRs from the sense G_4_C_2_ repeats (26, 29). To further demonstrate the contribution of frameshifting in the production of DPRs, we generated antibodies recognizing either a peptide (MELRSRAL) translated in frame with poly-GA from the CUG codon to the first G_4_C_2_ repeat (N-terminal antibody; N-ter GA) (26, 30) or a peptide (PGAGLRLRCLRP) translated in frame with poly-GP after the G_4_C_2_ repeats (C-terminal antibody; C-ter GP) (fig. S1A). Using human embryonic kidney (HEK293T) cell translation extracts, we performed in vitro translation of a capped transcript with 66 G_4_C_2_ repeats flanked by upstream and downstream sequences from the *C9ORF72* locus, incorporating hemagglutinin (HA), HIS, and FLAG tags in frame with poly-GA, poly-GP, and poly-GR, respectively (fig. S1, A to C). Immunoprecipitation with the N-terminal antibody (N-ter GA) was followed by immunoblotting with antibodies specific for the three tags located downstream of the repeats (fig. S1B). As expected, poly-GA was efficiently immunoprecipitated by the N-ter (GA) antibody (fig. S1C). Both poly-GP and poly-GR were also efficiently pulled down, indicating that they share the same N-terminal peptide and are chimeric molecules produced by a ribosomal frameshifting (fig. S1C). However, poly-GP and poly-GR were also observed after pull down with an antibody against HA (fig. S1, D and E), and we cannot rule out the possibility that this detection reflects in vitro coaggregation.

To further validate these results, we expressed (G_4_C_2_)_66_ repeats initiated by either a CUG translation start codon or a mutated CCG version in HEK-293T cells and performed immunoprecipitation in radioimmunoprecipitation assay (RIPA) buffer with the N-ter (GA) antibody (fig. S1, F to H). The CCG mutation reduced the production of DPRs in all three frames (fig. S1, F to H, left panels). Poly-GA, poly-GP, and poly-GR were all pulled down by the N-ter (GA) antibody in the CUG-66R transfected cells but not in the CCG-66R cells (fig. S1, F to H, right panels). Consistently, immunoprecipitation with an HA antibody, which is in frame with poly-GA, pulled down proteins recognized by the N-ter antibody (fig. S1I).

To further examine the effect of CUG→CCG mutation on the secondary structure of G_4_C_2_ transcripts, we used in-line probing and ribonuclease (RNase) S1 probing of CUG- and CCG-(G_4_C_2_)_66_ transcripts in vitro (figs. S2 to S4). In-line probing measures the intrinsic flexibility of an RNA molecule at each nucleotide (31), with flexible (usually unstructured or single-stranded) regions undergoing spontaneous cleavage in the presence of Mg^2+^ (figs. S2 and S3). The RNase S1 probing is used to map RNA single-stranded regions by selectively cleaving them with RNase S1 (32) (fig. S4). RNase S1 probing indicated that the CUG-to-CCG mutation induced structural changes limited to the vicinity of the mutation site, whereas the overall mRNA structure remained identical between the CUG- and CCG-(G_4_C_2_)_66_ transcripts (fig. S4). A single S1 cleavage at the position of the CUG→CCG mutation was lost, and another S1 cleavage was observed nine nucleotides downstream of the mutation (fig. S4). However, in-line probing that measures the intrinsic flexibility of an RNA molecule at each nucleotide did not reveal changes in the reactivity profile of the transcripts, including at the vicinity of the CCG mutation and G_4_C_2_ repeat (figs. S2 and S3).

## Mutation of the CUG translation start codon prevents expression of DPRs in vivo

To investigate the impact of the CUG initiation codon on DPR production in vivo, we then performed intracerebroventricular (ICV) injections in newborn mice of adeno-associated virus (AAV) constructs encoding 66 G_4_C_2_ repeats preceded either by the wild-type CUG initiation codon [CUG-(G_4_C_2_)_66_; CUG-66R] or by the mutant CCG codon [CCG-(G_4_C_2_)_66_; CCG-66R] (fig. S5A). Control littermates were generated by ICV injection of constructs with two G_4_C_2_ repeats [CUG-(G_4_C_2_)_2_; 2R]. Mice were aged for up to 15 months with longitudinal behavioral analyses and plasma collections. Tissues harvested at 10 or 15 months of age were used for histological and biochemical analyses to determine the effect of CUG→CCG mutation on the production of DPRs in vivo.

**Fig. 1.**
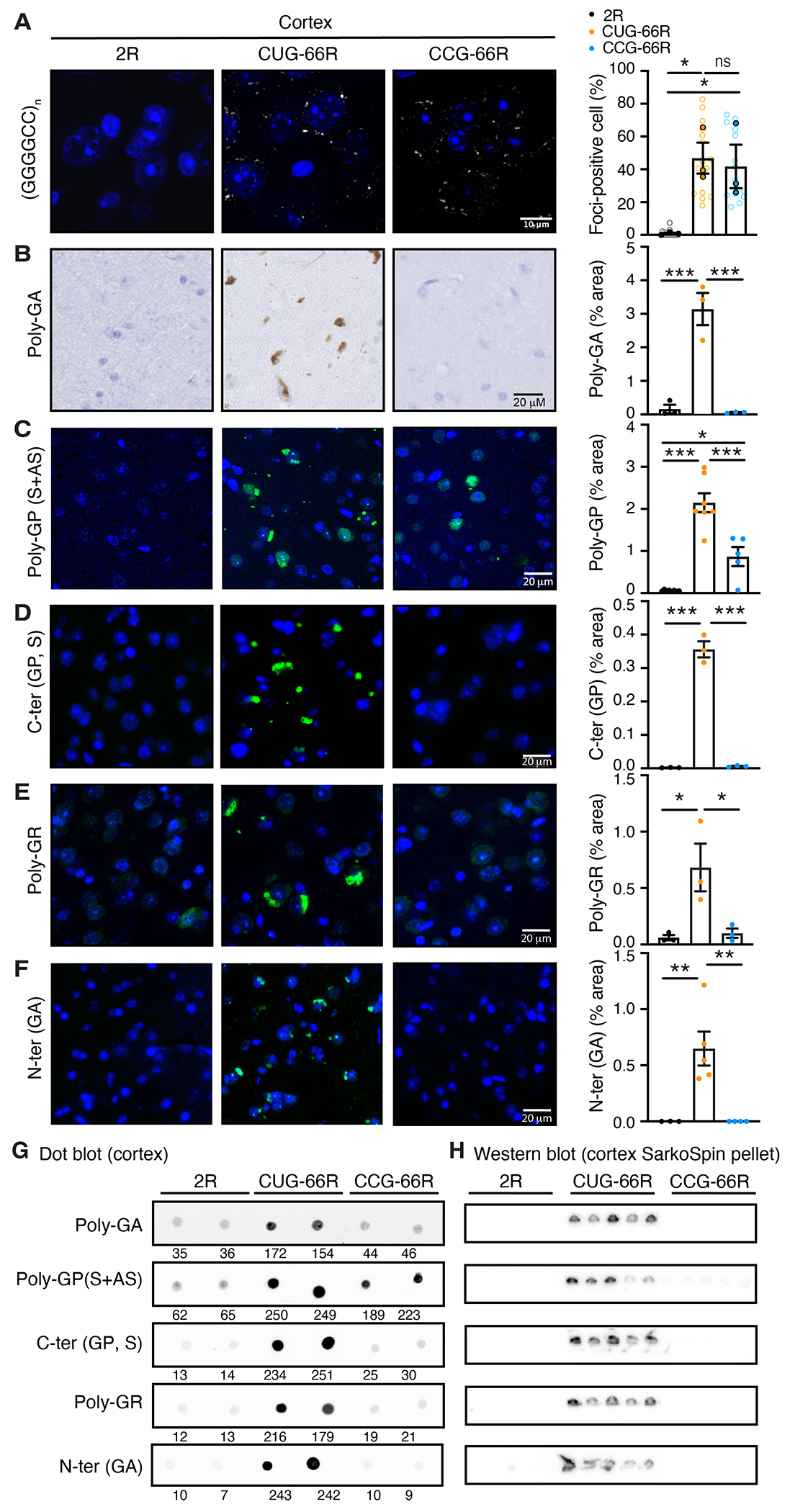
CUG initiation enables production of DPRs in all three frames from the *C9ORF72* repeat in (G_4_C_2_)_66_ mice. (**A**) FISH with probe targeting G_4_C_2_ repeats in the cortices of 10-month-old AAV-injected mice. The percentage of cells with foci was quantified. *N* = 3 mice per genotype; each open dot represents data from a field of view (FOV), and each solid dot represents the average value for each mouse. All FOVs were taken from two brain sections from the same mouse. Data are means ± SEMs. The statistical analysis was done with the averaged data (solid dot) using one-way analysis of variance (ANOVA) with Tukey’s multiple comparisons test. *P < 0.05. (**B** to **F**) Immunostaining of the cortices from 10-month-old mice with antibodies against poly-GA, poly-GP (sense + antisense), C-terminal peptide in frame with poly-GP (C-ter GP, sense), poly-GR, and N-terminal peptide in frame with poly-GA (N-ter GA). The percentages of area covered by DPR-positive pixels were quantified. *N* = 3 to 7 mice per genotype; each dot represents the average value from two brain sections from the same mouse. Data are means ± SEMs. Statistics by one-way ANOVA with Tukey’s multiple comparisons test. ns, not significant; *P < 0.05; **P < 0.01; ***P < 0.001. (**G**) Dot blot analysis of DPRs using lysate from cortex tissue of 15-month-old mice. *N* = 2 mice per genotype. The value below the blot represents the intensity of the dot. (**H**) Western blot analysis of the DPRs using SarkoSpin pellet fraction from cortex tissue of 15-month-old mice. *N* = 5 mice per genotype. Quantifications are provided in fig. S7.

First, we determined that the amounts of expanded RNAs forming RNA foci were not affected by the CUG→CCG mutation. Fluorescence in situ hybridization (FISH) (Fig. 1A) and reverse transcription quantitative polymerase chain reaction (RT-qPCR) (fig. S5B) demonstrated that the repeat-containing transcripts accumulated to similar amounts in CUG- and CCG-(G_4_C_2_)66 mice. In addition, expression of the endogenous mouse *C9orf72* gene was not altered upon ICV injections of AAV constructs (fig. S5C).

Then, we used immunostaining with antibodies recognizing the three sense DPRs, poly-GA, poly-GP, and poly-GR, as well as antibodies raised against N-terminal and C-terminal peptides (fig. S1A and Fig. 1, B to F). Although abundant aggregates were identified in the cortices of 10-month-old CUG-66R mice, mutating the CUG start codon blocked the expression of poly-GA and reduced the formation of poly-GP and poly-GR aggregates (Fig. 1, B to F, and fig. S6, A to C), providing further support for a frameshifting mechanism. The C-ter (GP) antibody, which detects only sense strand–derived poly-GP, labeled dense aggregates only in the cortices of CUG-66R mice (Fig. 1D). By contrast, the poly-GP antibody, which detects poly-GP from both sense and antisense transcripts, revealed both aggregated and diffuse nuclear staining in CUG-66R and CCG-66R mice (Fig. 1C and fig. S6, A to C), consistent with the CCG mutation affecting only sense poly-GP production. To further support a ribosome frameshifting mechanism, we developed a meso scale discovery (MSD) electrochemiluminescence detection technology, or immunoassay, which used the N-ter (GA) as the capture antibody and either poly-GA or poly-GP antibodies as detection antibodies (fig. S6D). In agreement with the in vitro immunoprecipitation results (fig. S1), this immunoassay detected both poly-GA and poly-GP in CUG-66R mice (fig. S6D).

We then used a combination of biochemical approaches to compare the expression of DPRs in the cortices from CUG-66R and CCG-66R mice. First, dot blot analysis of protein lysates from the cortices of 15-month-old mice detected DPRs using all five antibodies [poly-GA, poly-GP, C-ter (GP), poly-GR, and N-ter (GA)] in CUG-66R compared with 2R animals (Fig. 1G). All antibodies, except for the poly-GP antibody, showed reduction of DPRs in CCG-66R lysates to similar amounts as the 2R mice. Poly-GP antibody detected a lower expression of DPR in CCG-66R compared with CUG-66R lysates, consistent with the persistence of antisense poly-GP in CCG-66R mice.

To further evaluate the accumulation of DPRs in the insoluble and soluble protein fractions, we used SarkoSpin to isolate large protein complexes from mouse cortical tissue (30, 33, 34). The amounts of insoluble DPRs in the SarkoSpin pellets were significantly reduced in the CCG-66R mice compared with the CUG-66R mice (Fig. 1H and fig. S7, A to J). Low amounts of all DPRs were detected in the CCG-66R mice when increasing the exposure of the immunoblots except for the N-ter (GA), which is consistent with a small fraction of each DPR being produced from translation initiating at minor start sites within or outside of the repeats (fig. S7, K to O).

Next, we used the MSD immunoassay to measure the expression of soluble DPRs (30). Immunoassay measurements demonstrated significant reduction of poly-GA and poly-GR in the solubilized SarkoSpin pellets from the cortices of CCG-66R mice compared with the CUG-66R mice (fig. S8A). By contrast, soluble poly-GP, likely derived from the antisense strand, was detected in CCG-66R mice. This apparent increase of poly-GP may reflect that immunoassays do not measure large complexes and may underestimate the overall amounts of DPRs in CUG-66R mice, where poly-GA, poly-GP, and poly-GR mostly coaggregate (fig. S8B). Supporting this hypothesis, an immunoassay using total protein extracts from cortex failed to detect reduced poly-GR expression in CCG-66R mice (fig. S8C), whereas all of the other detection methods used found reduced amounts of poly-GR in these mice (Fig. 1, fig. S7, and fig. S8A). Together, these experiments demonstrate that the mutation of a single nucleotide in the CUG translation codon offers a powerful in vivo approach to unravel the respective contribution of DPRs and G_4_C_2_ repeat RNAs in driving disease-associated phenotypes.

## CUG→CCG mutation rescues behavioral deficits in *C9ORF72* (G_4_C_2_)_66_ mice

**Fig. 2.**
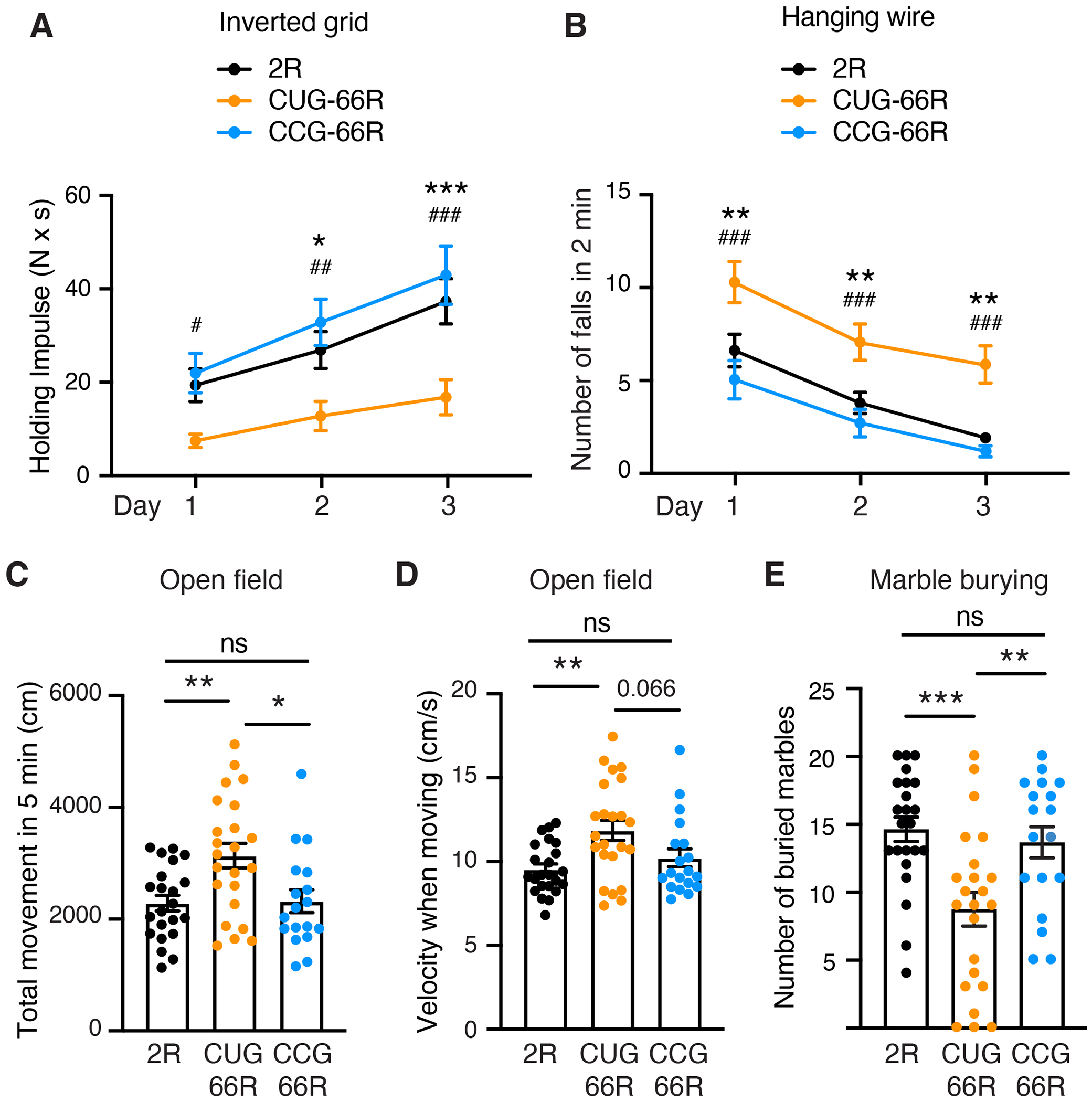
Reducing DPR amounts by mutating the CUG upstream of the repeat rescues behavior deficits in (G_4_C_2_)_66_ mice. (**A**) Inverted grid test performed in 9-month-old mice expressing (G_4_C_2_)_66_ with either a CUG or mutated CCG codon upstream of the repeat. Holding impulse = body weight (g) × 0.00980665 (N/g) × hanging time (s). *N* = 18 to 23 mice per genotype, both males and females. (**B**) Hanging wire test determined the number of falls within 2 min in 9-month-old mice. *N* = 14 to 20 mice per genotype. Data are means ± SEMs. Statistics by two-way ANOVA with Tukey’s multiple comparisons test. Asterisks indicate differences between 2R and CUG-66R; number signs (#) indicate differences between CUG-66R and CCG-66R. (**C** and **D**) Open field test in 8-month-old mice to determine the total movement (C) and mobility time (D) during the first 5 min of recording. (**E**) Marble burying assay was performed in 8-month-old mice. The number of buried marbles was counted after placing the mouse in the cage for 5 min. *N* ≥ 20 mice per genotype. Data are means ± SEMs. Statistics by one-way ANOVA with Tukey’s multiple comparisons test. *P < 0.05; **P < 0.01; ***P < 0.001.

To determine whether alleviating the production of DPRs without altering accumulation of repeat RNAs may affect behavioral abnormalities developed by *C9ORF72* mice, we subjected cohorts of 2R, CUG-66R, and CCG-66R animals to a battery of behavioral tasks. Substantial motor performance deficits were observed by inverted grid and hanging wire tests in both male and female CUG-66R mice compared with 2R mice, and these motor deficits were rescued in CCG-66R animals (Fig. 2, A and B, and fig. S9). In addition, the CCG mutation alleviated the hyperactivity phenotype of *C9ORF72* mice in an open field test (Fig. 2, C and D). CUG-66R mice traveled more distance at a faster pace compared with 2R animals during the first 5 min of testing, as reported in a previous study (35), whereas CCG-66R mice showed a reduction of the total distance traveled (Fig. 2C) and a trend toward slower velocity (Fig. 2D). Furthermore, using a marble burying test, we observed a de-crease of buried marbles in CUG-66R compared with 2R mice, a phenotype fully rescued in CCG-66R mice (Fig. 2E).

## CUG→CCG mutation rescues pathological hallmarks in *C9ORF72* (G_4_C_2_)_66_ mice

**Fig. 3.**
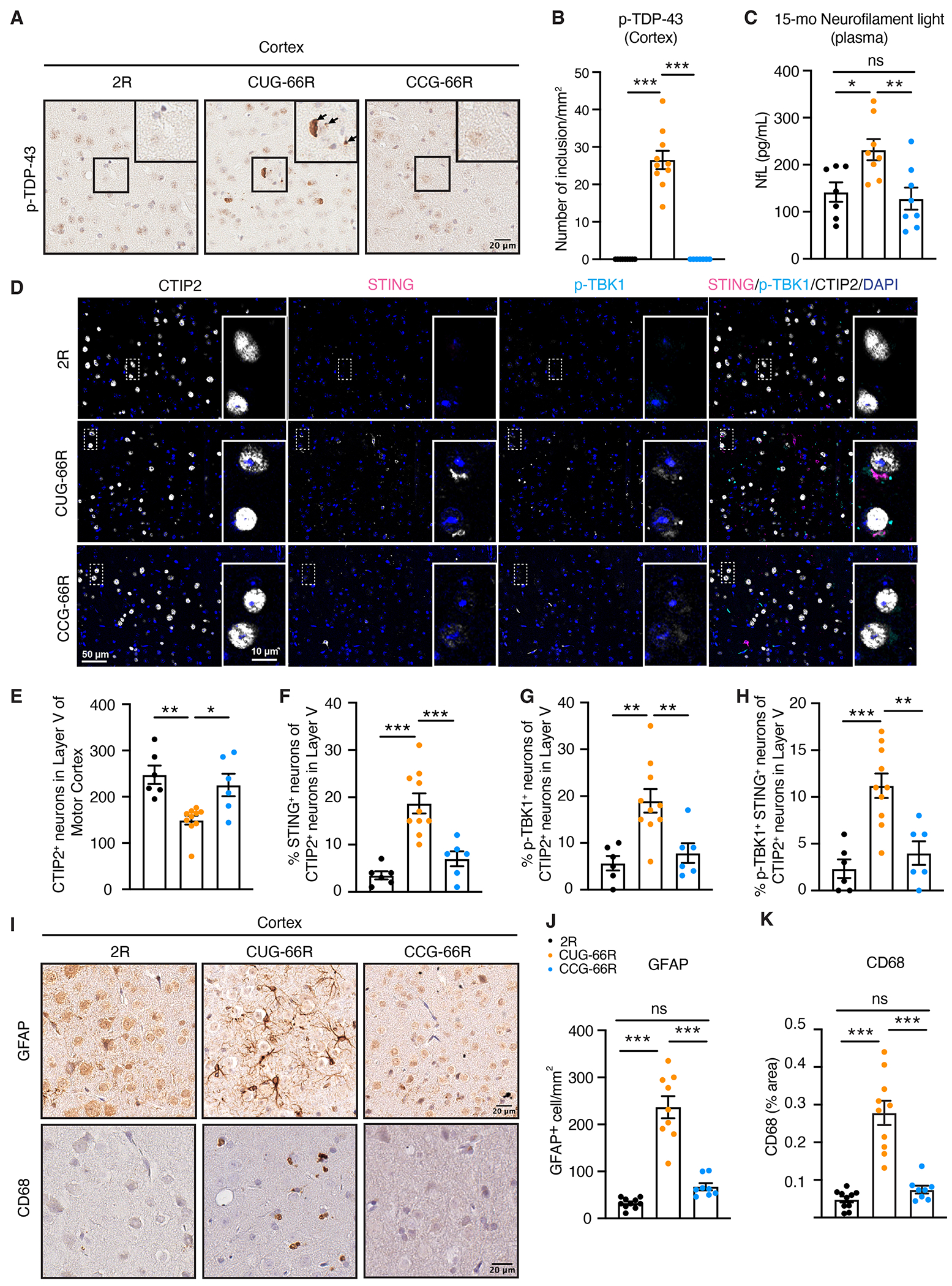
Reducing DPR amounts by mutating the CUG upstream of the repeat rescues pathological abnormalities in (G_4_C_2_)_66_ mice. (**A** and **B**) Immunohistochemistry staining with phosphorylated TDP-43 (p-TDP-43) antibody in the cortex of 15-month-old mice. The arrowheads in the insert point to p-TDP-43 aggregates. The number of inclusions per square millimeter was quantified in the whole cortex. *N* = 8 mice per genotype. Data are means ± SEMs. Statistics by one-way ANOVA with Tukey’s multiple comparisons test. ***P < 0.001. (**C**) NfL concentration in the plasma of 15-month-old mice. *N* = 6 to 8 mice per genotype. Data are means ± SEMs. Statistics by one-way ANOVA with Tukey’s multiple comparisons test. *P < 0.05; **P < 0.01. (**D** to **H**) Immunofluorescence staining of the layer V motor cortex in 15-month-old mice with antibodies against CTIP2, STING, and p-TBK1 (D). The number of CTIP2-positive neurons in layer V of the motor cortex (E), the percentage of CTIP2-positive cells with accumulation of STING (F), p-TBK1 (G), or both STING and p-TBK1 (H) were determined. *N* = 10 mice per genotype. Data are means ± SEMs. Statistics by one-way ANOVA with Tukey’s multiple comparisons test. *P < 0.05; **P < 0.01; ***P < 0.001. (**I** to **K**) Immunohistochemistry staining with antibodies against the astrocytes marker GFAP and activated phagocytic microglia marker CD68 in the cortex of 15-month-old mice expressing (G_4_C_2_)_66_ with either a CUG or mutated CCG codon upstream of the repeat. The number of GFAP-positive cells per square millimeter (J) and the percentage of area covered by CD68-positive pixel (K) were quantified. *N* = 8 mice per genotype. Data are means ± SEMs. Statistics by one-way ANOVA with Tukey’s multiple comparisons test. ***P < 0.001.

To further assess the impact of RAN translation inhibition in the AAV-*C9ORF72* mice, we first stained cortical sections from 15-month-old animals with a monoclonal p-TDP-43 antibody (36) and identified p-TDP-43 aggregates in CUG-66R mice, which were absent in CCG-66R mice across multiple brain regions (Fig. 3, A and B, and fig. S10A). The distribution of p-TDP-43 pathology highly correlates with the clinical phenotypes of *C9ORF72* patients, and the presence of p-TDP-43 was reported in AAV-(G_4_C_2_)_66/149_ mice and AAV-GFP-(GR)_200_ mice (35, 37–40). Our observation in CCG-66R mice supports a key contribution of DPRs in the disruption of TDP-43 homeostasis in *C9ORF72*-associated neurodegeneration. Ubiquitin is another pathological hallmark of *C9ORF72* disease (41), and Western blots from SarkoSpin pellets showed increased ubiquitin and polyubiquitin (K48) accumulation in the cortices of 15-month-old CUG-66R but not CCG-66R mice (fig. S10, B and C). Using a Simoa assay, we observed that CUG-66R displayed increased plasmatic amounts of neurofilament light (NfL), a clinical biomarker for neuronal injury (42), that was restored in both 12-month-old (fig. S10D) and 15-month-old CCG-66R mice (Fig. 3C). Then, we costained cortical sections with an antibody against CTIP2, which labels layer V motor neurons, and antibodies against STING and p-TBK1 that we recently showed to be altered in *C9ORF72* mice (43, 44) (Fig. 3D). Consistent with the increased plasmatic NfL, the number of CTIP2-positive neurons in layer V was reduced in CUG-66R compared with control 2R animals (Fig. 3, D and E), and remaining CTIP2-positive neurons exhibited abnormal accumulation of STING and p-TBK1 (Fig. 3, D and F to H). These phenotypes were reduced in CCG-66R mice, demonstrating that neurodegeneration and activation of STING and p-TBK1 were triggered by the production of DPRs (Fig. 3, D to H). Next, we stained cortical sections with antibodies against glial fibrillary acidic protein (GFAP) and CD68 that recognize activated astrocytes and microglia, respectively (Fig. 3I). The number of GFAP-positive cells (Fig. 3J) and the area positive for CD68 staining (Fig. 3K) were increased in the CUG-66R mice compared with 2R animals, consistent with the astrogliosis and microglia activation seen in postmortem tissues from patients with *C9ORF72*-ALS/FTD (45–47). Both were fully rescued by blocking RAN translation in the CCG-66R mice (Fig. 3, I to K).

## CUG→CCG mutation upstream of 158 repeats lowers DPR expression and mitigates phenotypes in (G_4_C_2_)_150_ mice

**Fig. 4.**
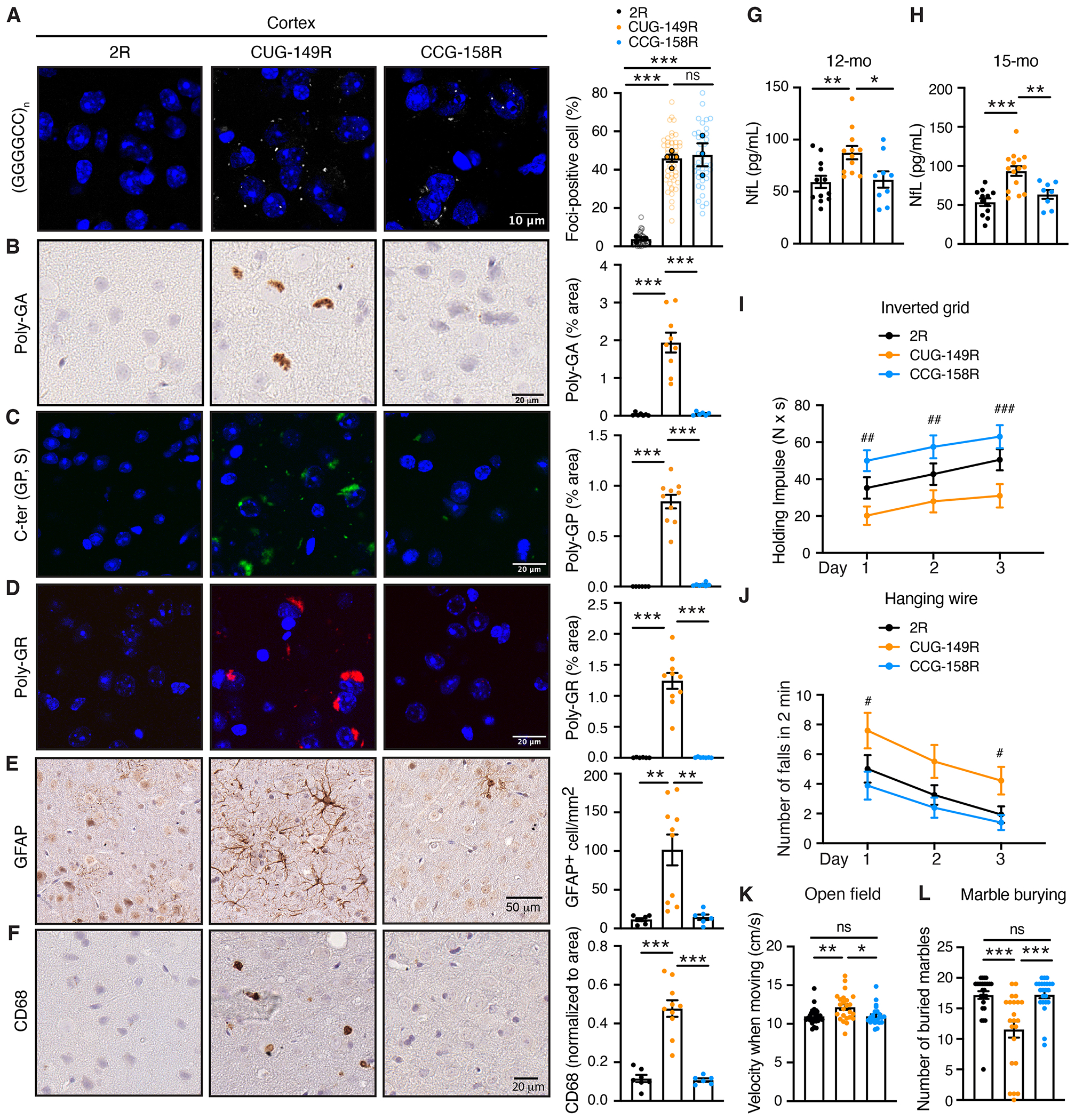
Rescue of pathological and behavioral changes in (G_4_C_2_)_149_ mice with CUG→CCG mutation. (**A**) FISH with probe targeting G_4_C_2_ repeats in the cortex of 18-month-old AAV-injected mice. The percentage of cells with foci was quantified. *N* = 3 to 4 mice per genotype; each open dot represents data from an FOV, and each solid dot represents the average value for each mouse. All FOVs were taken from two brain sections from the same mouse. Data are means ± SEMs. The statistical analysis was done with the averaged data (solid dot) using one-way ANOVA with Tukey’s multiple comparisons test. ***P < 0.001. (**B** to **D**) Immunostaining of the cortex from 17-month-old mice with antibodies against poly-GA, C-terminal peptide in frame with poly-GP (C-ter GP), and poly-GR. The percentages of area covered by DPR-positive pixel were quantified. *N* ≥ 6 mice per genotype. Each dot represents the average value from two brain sections from the same mouse. Data are means ± SEMs. Statistics by one-way ANOVA with Tukey’s multiple comparisons test. ***P < 0.001. (**E** and **F**) Immunohistochemistry staining with antibodies against the astrocytes marker GFAP and activated phagocytic microglia marker CD68 in the cortex of 15-month-old mice. The number of GFAP-positive cells per square millimeter (E) and the percentage of area covered by CD68-positive pixel (F) were quantified. *N* = 8 mice per genotype. Data are means ± SEMs. Statistics by one-way ANOVA with Tukey’s multiple comparisons test. **P < 0.01; ***P < 0.001. (**G** and **H**) NfL concentration in the plasma of 12-month-old (G) and 15-month-old (H) mice. *N* = 10 mice per genotype. Data are means ± SEMs. Statistics by one-way ANOVA with Tukey’s multiple comparisons test. **P < 0.01; ***P < 0.001. (**I**) Inverted grid test performed in 12-month-old mice. Holding impulse = body weight (g) × 0.00980665 (N/g) × hanging time (s). *N* ≥ 20 mice per genotype. (**J**) Hanging wire test determined the number of falls within 2 min in 12-month-old mice. *N* ≥ 20 mice per genotype. Data are means ± SEMs. Statistics by two-way ANOVA with Tukey’s multiple comparisons test. Asterisks indicate the differences between 2R and CUG-149R mice, and number signs (#) indicate differences between CUG-149R and CCG-158R mice. (**K**) Open field test in 12-month-old mice to determine the velocity when moving during the first 5 min of recording. (**L**) Marble burying assay was performed in 12-month-old mice. The number of buried marbles was determined after placing the mouse in the cage for 5 min. *N* ≥ 20 mice per genotype. Data are means ± SEMs. Statistics by one-way ANOVA with Tukey’s multiple comparisons test. *P < 0.05; **P < 0.01; ***P < 0.001.

Because RAN translation was shown to be influenced by the size of the repeat expansion (48, 49), we determined whether CUG→CCG mutation was beneficial in mice expressing a longer G4C2 repeat. We generated a mouse cohort expressing either 149 repeats downstream of the CUG codon [CUG-(G_4_C_2_)_149_; CUG-149R] or 158 repeats downstream of the CCG codon [CCG-(G_4_C_2_)_158_; CCG-158R] (fig. S11A). Despite the size of the repeat, mutating CUG into CCG in this independent cohort yielded similar results, supporting a major role for the CUG in the translation of the DPRs from all three reading frames. Indeed, as in the 66R mice (Fig. 1A and fig. S2B), the expression of repeat-containing RNAs (Fig. 4A and fig. S11B) and of endogenous *C9orf72* (fig. S11C) were not affected by the CUG-to-CCG mutation. Immunostaining for DPRs in the cortices of 6-month-old (fig. S11D) and 15-month-old (Fig. 4, B to D) mice revealed a substantial accumulation of DPRs in CUG-149R, which was markedly reduced in CCG-158R mice. Microglia and astrocytic activation were also reduced in CCG-158R mice compared with CUG-149R littermates (Fig. 4, E and F), and plasmatic NfL was similar in 2R and CCG-158R animals, whereas it was elevated in CUG-149R mice at both 12 and 15 months of age (Fig. 4, G and H). At the behavioral level, the CUG→CCG mutation improved motor deficits in both males and females (Fig. 4, I to L, and fig. S12). The holding impulse measured from an inverted grid assay in 12-month-old mice (Fig. 4I and fig. S12, A and B), as well as the number of falls from a hanging wire at 3 and 12 months of age (Fig. 4J and fig. S12, C to G), were rescued in CCG-158R animals. Abnormal velocity when moving in the open field test (Fig. 4K) and the number of buried marbles (Fig. 4L and fig. S12H) were also improved in the CCG-158R compared with CUG-149R mice.

## Genome editing of CUG to CCG in patient iPSC-derived motor neurons reduces DPR amounts

**Fig. 5.**
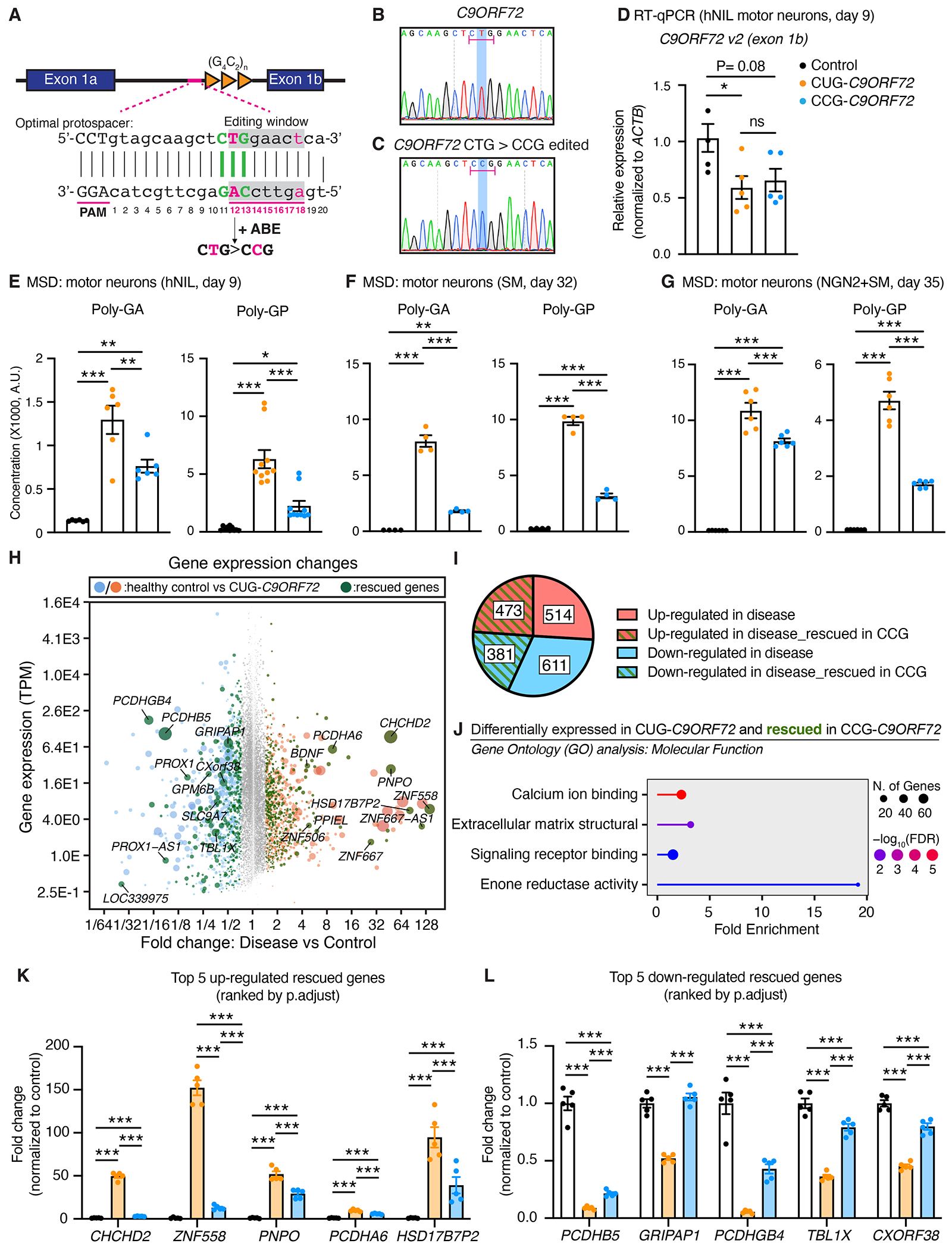
CUG→CCG base editing lowered DPR amounts and partially rescued gene expression in *C9ORF72* iPSC-derived motor neurons. (**A**) Schematic of the genome editing strategy using adenine base editor to mutate the CUG codon into CCG in *C9ORF72* iPSC lines. The A→G mutation (magenta) is at position 12 of the optimal protospacer. The base editing window of adenine base editor (ABE 6.3) is from position 12 to 18 (gray area). (**B** and **C**) Chromatograms of Sanger sequencing showing that the CUG codon (magenta underline) in the *C9ORF72* line (B) was precisely edited to CCG on both alleles (C). (**D**) RT-qPCR showing reduced expression of the *C9ORF72* transcript variant 2 in both CUG and CCG edited *C9ORF72* iPSC-derived motor neurons. *N* = 5 biological replicates per genotype from three independent differentiations. Data are means ± SEMs. Statistics by one-way ANOVA with Tukey’s multiple comparisons test. ***P < 0.001. (**E** to **G**) Immunoassay to determine the amounts of poly-GA and poly-GP in iPSC-derived motor neurons differentiated using expression of transcription factors (hNIL) for 9 days (E), small molecules for 32 days (F), and NGN2 + small molecules for 35 days (G). *N* > 6 replicates per genotype from two (poly-GA) to three (poly-GP) independent differentiations for hNIL neurons; *N* = 4 replicates per genotype from one differentiation for small molecules differentiated neurons; *N* = 6 replicates per genotype from one differentiation for NGN2+small molecules differentiated neurons. Data are means ± SEMs. Statistics by t test. *P < 0.05; **P < 0.01; ***P < 0.001. A.U., arbitrary units. (**H**) Volcano plot displaying differentially expressed genes in day 9 hNIL motor neurons. Genes with significant changes in mRNA expression between control and CUG-*C9ORF72* are represented by blue or red dots [log_2_fc > |0.5|, *P*_adjust_ < 0.05 by DESeq2 (109)]. Dot size is inversely proportional to the statistical significance. Green dots represent genes rescued in the CCG-*C9ORF72* (log_2_fc > |0.5|, *P*_adjust_ < 0.05 between CUG- and CCG-*C9ORF72*, or *P*_adjust_ > 0.05 between control and CCG-*C9ORF72*). The labeled genes are the top 10 rescued genes ranked by *P*_adjust_ on the up- or down-regulated side. Expression values are determined as transcripts per kilobase per million mapped reads (TPM). *N* = 5 biological replicates per genotype from three independent differentiation experiments. (**I**) Pie chart illustrating the proportion of up- and down-regulated genes between control and CUG-*C9ORF72*, and the proportion of genes rescued in CCG-*C9ORF72*. (**J**) GO molecular function terms enriched in rescued genes [ranked by false discovery rate (FDR) < 0.05]. The length of the lines represents the fold enrichment of each term, the size of the dots correlates with the number of genes in each term, and the color represents their FDR values. (**K** and **L**) Normalized TPM values for top five rescued genes in up-regulated (K) and down-regulated (L) transcripts between control and CUG-C9ORF72. Data are means ± SEMs; *N* = 5 biological replicates per genotype from three independent differentiation experiments. *P* values were derived from DESeq2 analysis. ***P < 0.001.

Because the number of repeats in *C9ORF72*-ALS/FTD patients can be hundreds to thousands (50), we sought to determine whether targeting the CUG codon could also reduce translation of DPRs and prevent phenotypes in iPSC-derived neurons. Whereas previous efforts had deleted the region preceding the repeat in iPSCs (51), we used a CRISPR adenine base–editing strategy (52) to precisely edit the CUG codon to CCG in a C9ORF72 patient-derived iPSC line (Fig. 5A). After single-cell sorting, clones were genotyped by Sanger sequencing (Fig. 5, B and C) and repeat-primed PCR (fig. S13A). We demonstrated that each iPSC clone expressed pluripotent markers (fig. S13B) and verified that none of the top potential off-target sites predicted by CCTop (52) were mutated in the selected clones (fig. S13, C to F).

We used a piggyBac transposase system to express doxycycline-inducible transcription factors; either neurogenin2 (NGN2) alone for differentiation into cortical neurons (53, 54) or NGN2 in combination with Islet-1 (ISL1) and LIM Homeobox3 (LHX3) (54–56) for differentiation into HB9-positive motor neurons (referred to herein as hNIL motor neurons) (fig. S13G). As previously reported in tissues from patients with *C9ORF72*-ALS/FTD (5, 57, 58) and iPSC-derived neurons (59–61), a reduction of the main *C9ORF72* transcript isoform (*C9ORF72* v2) was observed in both CUG and CCG *C9ORF72* hNIL motor neurons compared with control motor neurons (Fig. 5D). We then used immunoassay to determine the impact of CUG→CCG mutation on the translation of poly-GA and poly-GP in hNIL motor neurons. DPRs were detected in lysates from both CUG-*C9ORF72* and CCG-*C9ORF72* compared with control motor neurons but were reduced upon mutation of the start codon into CCG (Fig. 5E). Poly-GR was not detectable by immunoassay in any lines. Similar results were obtained using two independent methods of iPSC differentiation into motor neurons (62–64), with poly-GA and poly-GP amounts reduced upon CUG→CCG mutation (Fig. 5, F and G).

## CUG-to-CCG editing in *C9ORF72* iPSC-derived neurons partially rescues disease-associated transcriptional changes

RNA sequencing (RNA-seq) analysis was performed to determine the transcriptomic profiles of hNIL motor neurons derived from a healthy control and isogenic CUG- and CCG-*C9ORF72* iPSC lines (five replicates per genotype). Differential gene expression analysis revealed 987 up-regulated and 992 down-regulated transcripts in CUG-C9ORF72 motor neurons compared with control [adjusted P value (*P_adjust_*) < 0.05 and log_2_ fold change (log_2_fc) > 0.5; Fig. 5H, fig. S14A, and table S1]. Forty-three percent of the genes altered in CUG-*C9ORF72* motor neurons were rescued by the CUG-to-CCG editing (Fig. 5, H and I; fig. S14B; and table S1). Gene ontology (GO) analysis of the rescued genes revealed an enrichment for channel-related genes that were down-regulated in the CUG-*C9ORF72* and rescued in the CCG-*C9ORF72* motor neurons (Fig. 5J, fig. S14C, and table S2). A significant enrichment was also found for genes related to the extracellular matrix (ECM) that were up-regulated in CUG-*C9ORF72* and rescued in the CCG-*C9ORF72* motor neurons (Fig. 5J, fig. S14D, and table S2). This is consistent with the previous observation of up-regulated ECM genes in (GR)_400_ and (PR)_400_ knock-in mice and iPSC-derived motor neurons (65). The top rescued genes (ranked by the adjusted *P* value between control and CUG-*C9ORF72*) included several protocadherins (PCDHA6, PCDHB5, and PCDHGB4); the mitochondria-associated gene CHCHD2; and genes involved in neuronal signaling, such as *GRIPAP1*, *PNPO*, and *TBL1X* (Fig. 5, K and L).

## CUG-to-CCG editing in *C9ORF72* iPSC-derived neurons rescues disease-associated cellular phenotypes

**Fig. 6.**
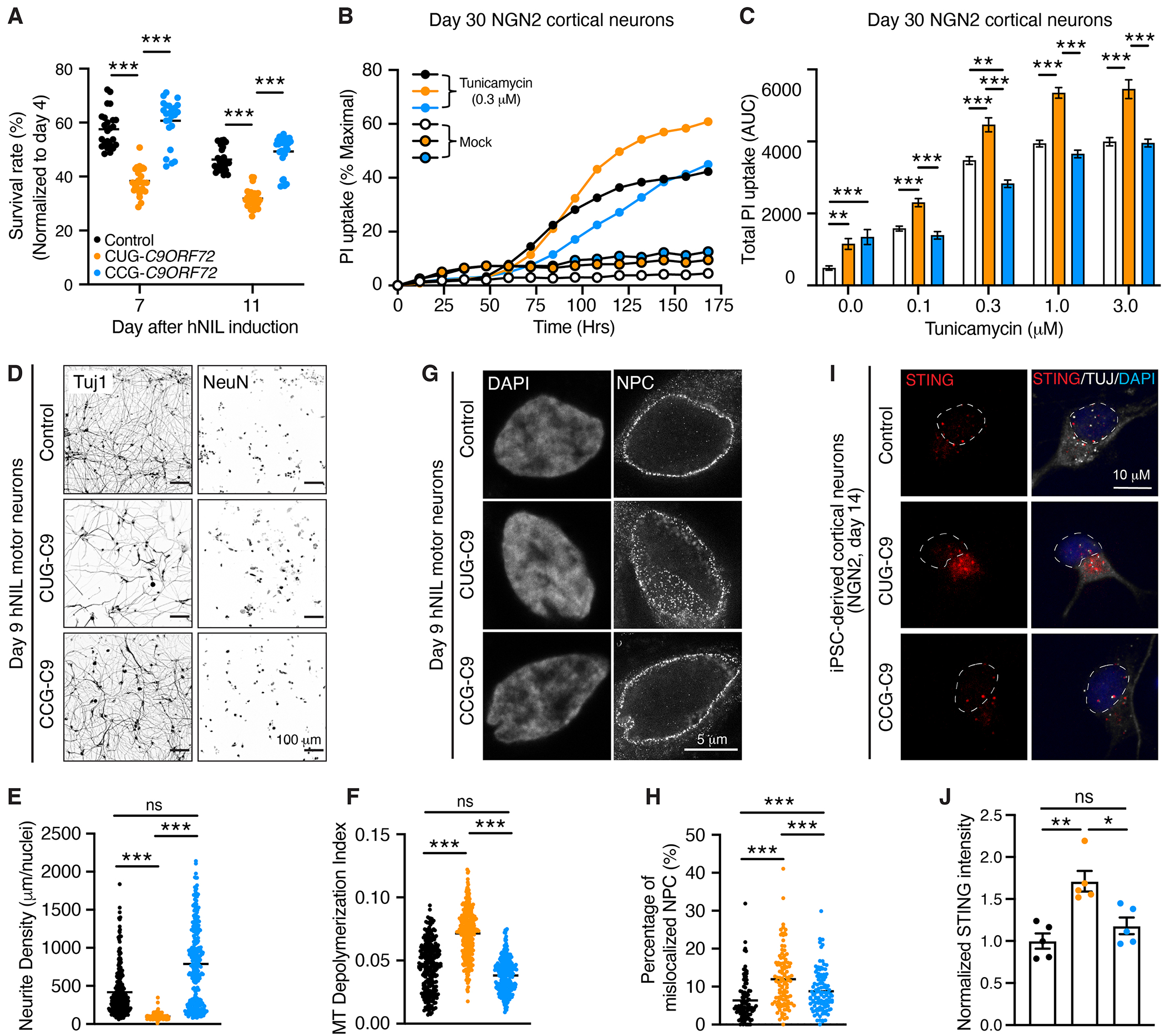
CUG→CCG editing in the *C9ORF72* neurons ameliorates disease-related phenotypes. (**A**) Longitudinal live-cell imaging to determine the number of alive motor neurons on day 4, day 7, and day 11 after induction of hNIL. *N* > 24 wells per genotype from three (day 7) to four (day 11) independent differentiations; the number of alive cells per well on days 7 and 11 were normalized to the alive cells in the same well on day 4. The solid dots represent the averaged value from individual differentiation. Statistics by two-way ANOVA with Tukey’s multiple comparisons test. ***P < 0.001. (**B**) NGN2 neurons were incubated in PI-containing medium and treated with 0.3 μM tunicamycin. Images were acquired every 12 hours over 7 days (*N* = 16 wells per genotype; three images per well per time point). Data are means ± SEMs. (**C**) Area under the curve (AUC_168 hours_) measurements of PI uptake in NGN2 neurons treated with varying doses of tunicamycin. Data are means ± SEMs. Statistics by two-way ANOVA with Tukey’s multiple comparisons test. **P < 0.01; ***P < 0.001. Corresponding curve graphs for 0.1 μM, 1.0 μM, and 3.0 μM tunicamycin are shown in fig. S15. (**D** to **F**) TUJ1 and NeuN staining of day 9 hNIL motor neurons (D) was used to assess the neurite density (E) and microtubule (MT) depolymerization index (F) across three cell lines. *N* = 20 wells per genotype in total from two independent differentiation; each dot in the graphs represents one FOV, and 9 FOVs were taken per well. Statistics by Brown-Forsythe and Welch ANOVA test. ***P < 0.001. (**G** and **H**) STED super-resolution microscopy of NPC staining in day 9 hNIL neurons was used to assess NPC localization. A total of *N* = 100 nuclei per genotype were analyzed, collected from six wells across two independent differentiations. Each dot in the graph represents one nucleus. Statistical significance was determined by Kruskal-Wallis test. ***P < 0.001. (**I**) Immunostaining with antibodies against STING and the neuronal marker TUJ1 in D14 (day 14 after differentiation) NGN2 cortical neurons. (**J**) The intensity of STING signal in TUJ1-positive neurons was quantified, and the data were normalized to the control line. *N* = 5 wells per line from two independent differentiations; each dot represents the averaged value from 15 to 25 FOVs per well. Data are means ± SEMs. Statistics by one-way ANOVA with Tukey’s multiple comparisons test. *P < 0.05; **P < 0.01.

The impact of editing CUG to CCG and reducing DPRs in iPSC-differentiated neurons was also assessed using live-cell imaging. We first demonstrated that mutation of the start codon improved the survival of hNIL motor neurons, with significant differences (*P* < 0.001) between isogenic CUG-*C9ORF72* and CCG-*C9ORF72* neurons (Fig. 6A). We then subjected NGN2 cortical neurons to increasing doses of tunicamycin (0.1 to 3 μM)—which induces protein misfolding and endoplasmic reticulum (ER) stress—and measured propidium iodide (PI) uptake over time to assess cell viability (Fig. 6, B and C, and fig. S15). Tunicamycin treatment caused a marked increase in PI uptake in all lines with an increase in CUG-*C9ORF72* neurons com-pared with both CCG-*C9ORF72* and control neurons (Fig. 6, B and C, and fig. S15). In addition, beta-III-tubulin (TUJ1) staining demonstrated reduced neurite density and increased microtubule depolymerization index in CUG-*C9ORF72* motor neurons compared with control (66, 67), with both phenotypes being restored in the CCG-*C9ORF72* motor neurons (Fig. 6, D to F). Because disruption of the nucleocytoplasmic transport contributes to *C9ORF72*-associated ALS and FTD pathogenesis (68–70), we stained hNIL motor neurons with an antibody against the nuclear pore complex (NPC). Using stimulated emission depletion (STED) superresolution microscopy, we observed an increased mislocalization of NPCs—likely resulting from abnormal invaginations of the nuclear membrane—in the CUG-*C9ORF72* neurons compared with both CCG-*C9ORF72* and control motor neurons (Fig. 6, G and H). Finally, as observed in cortex of CUG-66R mice (Fig. 3, D and F), iPSC-derived cortical neurons from patients with *C9ORF72*-ALS/FTD displayed increased accumulation of cytoplasmic STING (43), a phenotype that was rescued by mutating the translation codon into CCG (Fig. 6, I and J). Collectively, these experiments demonstrate that editing the CUG start codon reduced DPR expressions and rescued disease-related phenotypes in patient-derived iPSC neurons.

## Discussion

*C9ORF72*-related ALS and FTD are driven by complex mechanisms, with toxicity proposed to stem from the concurrent accumulation of RNA foci and aberrant DPRs (6–9). Understanding the specific contribution of repeat RNAs versus DPRs is crucial for designing therapeutic interventions; however disentangling accumulation of repeat RNAs from RAN translation has been challenging. To investigate the isolated role of repeat RNA or DPRs, four main approaches have been attempted to selectively introduce repeat RNAs with no or limited DPRs in nonmammal animal models or cultured neurons: (i) expressing an RNA-only construct containing G_4_C_2_ repeats interrupted with stop codons every 12 repeats (19, 20, 25, 63, 71, 72), (ii) overexpressing short G_4_C_2_ repeats (24, 73), (iii) flanking repeats with intronic and exonic sequences (22), and (iv) using short-term antisense oligonucleotide (ASO) treatments in a time frame where reduced RNA foci is not yet accompanied by reduced expression of DPRs (24, 63, 71). *Drosophila* models overexpressing the RNA-only construct did not exhibit survival deficits even though RNA foci sequestered the RNA binding protein Glorund (Glo) (19, 20). Glo is the *Drosophila* ortholog of hnRNP H, which is known to interact with *C9ORF72* repeat RNAs (14, 15, 74, 75), supporting that repeat RNAs alone play a limited role in driving disease progression. On the other hand, cultured neurons or zebrafish overexpressing a short repeat sequence, a repeat flanked with intronic and exonic sequence, or an RNA-only construct displayed disease-related phenotypes, and treatment with ASOs for a short term was beneficial despite the remaining accumulation of DPRs (22, 24, 25, 72, 73). This may arise from limitations in DPR detection methods, which could underestimate their contribution to disease, warranting caution when attributing phenotypes solely to repeat RNAs. In this study, we leveraged our recent finding that a noncognate translation start codon upstream of the repeat serves as a major site to initiate the translation of all three DPRs from the sense direction through a frameshifting mechanism (26). By introducing a single-nucleotide mutation in this translation start codon (CUG→CCG), we successfully suppressed DPR synthesis without interfering with the secondary structures of repeat RNAs in both *C9ORF72* mice and *C9ORF72* iPSC-derived motor neurons. The CUG→CCG mutation rescued behavioral deficits and pathological changes in *C9ORF72* mice as well as survival deficits and molecular phenotypes in cultured neurons. These findings align with recent work showing that neuronal overexpression of a CUG→UAG G_4_C_2_ repeats construct in *Caenorhabditis elegans* also improved survival compared with diseased worms (76). Collectively, our results provide direct support that toxicity from DPRs is a more functionally relevant contributor to disease progression than G_4_C_2_ repeat RNA toxicity. Evidence supporting a predominant role for toxic peptides rather than CGG repeat RNAs was also reported in fragile X–associated tremor/ataxia syndrome (FXTAS) (77). Although the AAV-(G_4_C_2_)_n_ mouse models provide valuable insights and yield reproducible behavioral and pathological phenotypes, we acknowledge that this virus-mediated overexpression approach does not fully recapitulate all *C9ORF72*-ALS/FTD disease mechanisms. Therefore, future studies using a mouse knock-in model with long G_4_C_2_ repeats inserted in a humanized *C9orf72* intron would provide a powerful approach to evaluate the effects of the CUG-to-CCG mutation.

The mechanisms behind the synthesis of proteins from expanded repeats remain elusive and seem to be largely context dependent, with surrounding sequences and cell type–specific factors influencing the production of aberrant peptides (13, 49, 78). We and others have used in vitro translation and expression of repeat-containing constructs in cell lines to identify a CUG codon as a key translation start site of the *C9ORF72* G_4_C_2_ repeat, and we showed that this start codon enabled the translation of DPRs from all three frames through ribosomal frameshifting (26–28). In this study, we provide evidence that sense poly-GP and poly-GR are also primarily generated from the CUG codon in vivo and in patient iPSC-derived neurons. This frameshifting event is further supported by the reported accumulation of chimeric DPRs in vitro and chimeric GA-GP in *C9ORF72* patient brains (29, 79), and a similar mechanism was reported in FXTAS (80). Since the discovery of RAN translation in 2011 (49), compelling evidence has demonstrated that repeat-containing transcripts can be translated in the absence of a canonical, or near-cognate, start codon (27, 81–88). In this work, we leverage the observation that mutation of a CUG codon preceding the *C9ORF72* repeat reduced production of DPRs from the three frames to test the relative contribution of DPRs versus RNA toxicity. We observed that the mutation induced localized structural change at the proximity of the mutated codon without affecting the repeat expansion structure. Although the change is limited, it will be important to determine whether the CUG-to-CCG mutation is associated with posttranscriptional RNA modifications or altered protein-RNA interactions that may modulate translation efficiency. The amounts of poly-GP and poly-GR expression varied across multiple cell types when different CUG-mutated repeat-containing constructs were expressed (26, 27, 76, 79, 89, 90), which emphasizes the importance of using complementary models, including *C9ORF72* patient-derived neurons. A previous study observed a reduction in poly-GA amounts, whereas poly-GP and poly-GR remained largely unaffected, after deleting 86 base pairs of the intron preceding the repeats in *C9ORF72* patient-derived neurons (51). In the current study, we precisely edited the CUG codon in patient iPSCs and found that both poly-GA and poly-GP amounts decreased up to 70% in the CUG→CCG edited motor neurons (Fig. 5, E to G). Although the amounts of poly-GA and poly-GP did not return fully to control amounts—suggesting the presence of alternative translation initiation mechanisms and consistent with poly-GP being also generated from the antisense strand—our in vitro and in vivo results highlight the critical role of the CUG codon in the translation of *C9ORF72* DPRs. Because the DPRs are not equally toxic (18), future studies to elucidate the precise mechanisms of frameshifting will be valuable. The recent discontinuation of two clinical trials using ASOs targeting *C9ORF72* sense transcripts underscores the importance of pursuing alternative strategies (91–93). We provide evidence that focusing on the production of DPRs through frameshifting or other mechanisms rather than reducing C9ORF72 transcripts may be beneficial.

## Materials and Methods

### Generation of C9ORF72 mice

All animal procedures were performed in accordance with the National Institutes of Health Guide for Care and Use of Experimental Animals and approved by the MGH Institutional Animal Care and Use Committee (protocol number 2015N000229).

AAV viruses were provided by L. Petrucelli (Mayo Clinic, Jacksonville). Briefly, the (G_4_C_2_)_2_, (G_4_C_2_)_66_, or (G_4_C_2_)_149/158_ repeats (with either a native CUG start codon or mutated CCG codon), along with 119 and 100 base pairs of the 5′ and 3′ regions flanking the repeat in the human *C9ORF72* gene, respectively, were inserted into an AAV expression vector and purified. AAV injections into the lateral cerebral ventricles of postnatal day 0 (P0) C57BL/6J pups were performed as previously described (35, 40) using 2 μl (1 × 10^10^ genomes per microliter) of AAV9-2R or AAV9-CUG/CCG-(G_4_C_2_)_66/150_ solution per cerebral ventricle. AAV injections and all subsequent in vivo experiments were conducted using cohorts with approximately equal numbers of male and female animals. Data obtained from both sexes are presented together, except when a significant difference between males and females was observed.

In rare cases, ICV injections led to the development of hydrocephalus, a complication that is independent of the genotype. Mice presenting hydrocephalus were excluded from the behavioral and pathological analyses.

### Mouse behavioral tests

All mice were acclimated to the behavioral room for 30 min before the tests. All behavioral equipment was cleaned with 70% ethanol between each mouse. All mice were placed back to the home cage and home room after the experiments. Investigators remained blinded to the genotypes throughout the behavioral and pathological analyses. Sample size for each cohort was determined based on our previous behavioral characterization of the model (10, 30, 35, 40).

#### Hanging wire test:

A 2-mm-thick wire tied to two vertical stands, ~55 cm apart, was maintained 35 cm above a layer of 5 cm bedding material to prevent injury when an animal fell from the wire. Upon grasping the wire, the number of times the mouse fell within a 2-min time period was recorded. In each session, the procedure was repeated three times with ~5 min between each assessment of holding time

#### Inverted grid:

The test was performed as described (94) with minor modifications. Each mouse was placed on a 30 cm × 45 cm wire grid for 5 s to accommodate, then the grid was inverted and held 35 cm over a foam pad for 5 min. Each of these holding periods began with all four paws of the mouse grasping the grid. The wire grid hanging time was defined as the amount of time that it took the mouse to fall from the inverted grid. In each session, the procedure was repeated three times with ~5 min between each assessment of holding time. The mouse body weight was obtained shortly before the test. The physical impulse (holding impulse) is the hanging time multiplied by the gravitational force of the mouse [body weight (g) × 0.00980665 (N/g) × hanging time (s)].

#### Open field test:

Each mouse was placed in the center of a 40 cm × 40 cm × 35 cm (width × length × height) arena and allowed to freely move for 15 min. The camera overhead the arena recorded the movement of the mice and EthoVision XT software was used to track and analyze their activities. Anxiety levels of the mice was measured by the total movement and velocity when moving in the first 5 min in the arena (95).

#### Marble burying test:

As described (96), each mouse was placed in a cage containing bedding that is 5 cm in depth, with 20 marbles arranged equidistantly in a 4 × 5 arrangement. After 20 min, the mouse was returned to the home cage and the number of marbles buried (at least 2/3 covered by bedding) was determined. After each testing period, the marbles were cleaned with 10% bleach, rinsed with water, and left to dry for 5 min before the next test.

### Blood collection and plasma NfL measurement

One hundred to 150 μl of blood per mouse was collected by facial vein sampling. To have a duplicate for each sample measurement, we performed two blood collections for each animal at 2 weeks interval and pooled the samples from the same mouse for NfL measurement. Blood was collected in BD Microtainer K2E tubes (365974) and centrifuged at 5000 rpm for 5 min at 4°C. After centrifugation, the plasma was immediately transferred into a clean Eppendorf tube. Samples were maintained at 2° to 8°C while handling and stored at −80°C. Simoa NF-light Advantage Kit (103186) was used to measure the NfL concentration with Quanterix HD-X Automated Immunoassay Analyzer.

### Immunostaining and quantification of DPRs, CD68, GFAP, CTIP2, p-TBK1, and STING

After perfusion with ice-cold phosphate-buffered saline (PBS), half brains of the mice were fixed in 4% paraformaldehyde (Electron Microscopy Sciences, 15713) for 24 hours, embedded in paraffin and sliced into 5-μm-thick sections (sagittal) and mounted on glass slides. Tissue sections were deparaffinized in CitriSolv Hybrid (Decon Labs, Inc., 1601H) and rehydrated through a series of ethanol solutions. Antigen retrieval was performed by autoclaving in citrate-based antigen unmasking solution (Vector Laboratories, H-3300) for 15 min. After 3% hydrogen peroxide treatment for 30 min, the slides were immersed with the blocking buffer (10% normal goat serum in 0.1% Triton X-100) for 1 hour. Then, tissues were immunostained with primary antibodies (table S3) in blocking buffer overnight at 4°C.

For immunohistochemistry, slides were then washed three times with 1xTBS with 0.1% TritonX-100 and incubated with HRP-conjugated secondary antibody for 1 hour at room temperature (DAKO K4003 or DAKO K4001). Slides were subsequently washed with another round of 1xTBS 0.1% Triton X-100, incubated with DAB substrate (DAKO K3468) solution for 10 min at room temperature and washed three times in distilled water. Sections were counterstained with hematoxylin (RICCA, 353032), dehydrated through a series of ethanol and xylene washes, and coverslipped with mounting media (Epredia, 4112). Images were taken with Nanozoomer, 40X magnification (Hamamatsu Photonics). For poly-GA and CD68 staining, the percentage of positive pixel per hemisphere was analyzed by Qupath. For GFAP, the number of GFAP positive was counted manually by investigators blinded to the genotypes. For immunofluorescence, slides were washed three times with PBS (with 0.1% Triton X-100) and incubated with Alexa Fluor Secondary Antibodies (Thermo Fisher Scientific) for 2 hours at room temperature. Slides were subsequently washed with PBS (0.1% Triton X-100), then incubated with 4′,6-diamidino-2-phenylindole (DAPI) (Invitrogen P36935) for 15 min and 0.1% Sudan black (Fisher BioReagents, BP109-10) for 10-s to quench the autofluorescence of lipofuscin. The slides were washed with water and coverslipped with antifade reagent with DAPI (Invitrogen, P36931). For DPRs, images were taken with Nikon C2 confocal microscope (60X magnification). Six images per section were taken from the cortex region with two sections analyzed for each mouse. The area of positive pixel was counted automatically by using FIJI. For STING, CTIP2, and p-TBK1, confocal images were taken using a 20X objective on a Zeiss LSM 900 Confocal microscope and the whole cortex were scanned. The quantification was done in the layer V cortex and custom analysis scripts (with FIJI) are available at https://github.com/wainger-lab/STING (97).

### Immunostaining and quantification of p-TDP-43

The staining was performed as previously described (40). Briefly, paraffine embedded sagittal brain sections (5 μm) were deparaffinized in xylenes (Honeywell, 534056) and then rehydrated in a graded series of ethanol. After washing in water, slides were immersed in antigen retrieval buffer (10 mM sodium citrate, 0.05% Tween-20, pH = 6.0) and steam for 30 min. Slides were cooled down for 15 min, and then gently ran under distilled water for 15 min. The slides were immersed into Dako Dual Endogenous Enzyme Block (DAKO, S2003) for 5 min, washed three times in PBS and then incubated with blocking buffer (2% normal goat serum in PBS) at room temperature for 1 hour, before incubation with p-TDP-43 antibody (40) at 4°C overnight. The slides were then washed three times in PBS and incubated with biotinylated goat anti-rabbit secondary antibody (Vector, PK-6101) at room temperature for 2 hours. The slides were washed again with PBS and incubated with ABC complex at room temperature for 30 min, before washing with PBS. Slides were developed with DAB buffer (ACROS Organics, 1 μl of hydrogen peroxide per 5 ml of DAB) for 5 min, then rinsed in distilled water, counterstained with hematoxylin and dehydrated through a series of ethanol washes and xylene. Finally, they were coverslipped with Cytoseal mounting media (Thermo Fisher Scientific) and scanned with a ScanScope AT2 (Leica Biosystems) at 40× magnification. The number of p-TDP-43 inclusions was quantified manually by investigators blinded to the genotypes.

### RNA FISH

The staining was performed as previously described with minor modifications (11, 98). After perfusion with ice-cold RNase-free PBS, half brains of the mice were fixed in 4% paraformaldehyde for 24 hours, then dehydrated in 15% sucrose (in PBS) for 1 day, and changed to 30% sucrose on the second day. Tissue was embedded in Tissue-Tek O.C.T. compound (Sakuraus, 4583), sectioned (14 μm) with cryotome and mounted on glass slides. LNA DNA probe targeting sense G_4_C_2_ repeats was purchased from Exiqon (cat. no. 339500 and GeneGloge ID: LCD0175083-BKN; table S4). Sections were permeabilized in PBS with 0.2% Triton X-100 for 10 min at room temperature, then washed twice with PBS. After dehydrating the slides with a series of ethanol and air dry, they were pre-heated with hybridization buffer [10% w/v Dextran sulfate (Millipore, S4030), 10 mM Ribonucleoside Vanadyl Complex (NEB, S1402S), 2x SSC pH 5 (Invitrogen, 15557044), 50 mM Sodium Phosphate Buffer pH 7.2 (teknova, P2072), 50% fresh and deionized formamide (IBI Scientific, 72024)] for 30 min at 66°C. The slides were hybridized with 60 nM denatured probe at 66°C overnight. The next day, they were washed with 2x SSC (0.1% Tween-20) for 5 min at room temperature, then three times with 0.2% SSC at 60°C for 10 min each. DAPI was used to stain nuclei and the autofluorescence of lipofuscin was quenched by dipping the slides in 0.1% Sudan Black. Slides were washed with DEPC-treated water and dehydrated with a series of ethanol solutions and then mounted with antifade reagent with DAPI (Invitrogen, P36935). Images were taken with Nikon C2 confocal microscope (100X magnification). Five images were taken from each animal, and the number of foci-positive cells was counted manually by investigators blinded to the genotypes.

### Dotblot

Tissues were homogenized in TE buffer [50 mM Tris HCl pH 7.4, 50 mM NaCl, 1 mM ethylenediaminetetraacetic acid (EDTA)] with the addition of fresh Protease Inhibitor (Roche, 4693116001) and Phosphatase Inhibitor Cocktail 2 (Sigma-Aldrich, P5726). 100 μl protein extraction buffer (50 mM Tris HCl pH 7.4, 300 mM NaCl, 5 mM EDTA, 0.1% Triton-X-100 with 3% SDS), 1 μl Benzonase nuclease HC (Millipore, 71206) and 1.4 μl 500 mM MgCl_2_ were added to each 50 μl homogenate. Then each sample was sonicated (Fisher Scientific, Sonic Dismembrator, Model 100, program 3). For dot blotting, samples were loaded on a 0.2 μm nitrocellulose membrane (BIO-RAD, 1620112) and air dried for 1 hour at room temperature. Samples were then blocked with 5% bovine serum albumin (BSA) in TBST with 0.2% Tween-20 for 1 hour at room temperature and washed four times in TBST with 0.2% Tween-20 for 5 min before incubation with primary antibodies (table S3) at 4°C overnight. On the following day, the membrane was washed, incubated with secondary antibodies for 2 hours at room temperature, and washed four times with TBST. Images were taken with the ChemiDoc MP Imaging System (BIO-RAD). The dot signal intensity was measured with FIJI.

### SarkoSpin

The SarkoSpin was performed as previously reported (33, 34). Briefly, frozen mouse cortex tissue was homogenized at 20% (w/v) in homogenization solubilization (HS) buffer: 10 mM Tris, pH 7.5, 150 mM NaCl, 0.1 mM EDTA, 1 mM dithiothreitol, complete EDTA-free protease inhibitors (Roche, 4693116001) and PhosSTOP phosphatase inhibitors (Roche, 4906845001). Then, the samples underwent three cycles of 30-s homogenization in tubes filled with a combination of ceramic beads measuring 1.4 and 2.8 mm in diameter, using a Minilys device (Bertin) operating at maximum speed, with cooling on ice between each cycle. After homogenization, the samples were divided into 150 μl aliquots, that were adjusted to equal protein concentration in a final volume of 200 μl using HS buffer. Subsequently, the samples were diluted 1:1 in HS buffer supplemented with 4% (w/v) N-lauroyl-sarcosine (sarkosyl, Sigma-Aldrich), 2 U/μl Benzonase (Novagen, 707463), and 4 mM MgCl_2_, resulting in a final volume of 400 μl with concentrations of 2% sarkosyl, 1 U/μl Benzonase, and 2 mM MgCl_2_. The SarkoSpin solubilization step was carried out by incubating the samples at 37°C with constant shaking at 600 rpm (using a Thermomixer, Eppendorf) for 45 min. After solubilization, the brain homogenates (400 μl) were further diluted by adding 200 μl of ice-cold HS buffer containing 0.5% (w/v) sarkosyl before centrifugation at 21,200*g* on a benchtop centrifuge (Eppendorf) for 30 min at room temperature. Supernatants (~600 μl) were collected in new tubes, and pellets were resuspended in the desired volume of HS buffer containing 0.5% (w/v) sarkosyl for Western blot and immunoassay.

For SDS–polyacrylamide gel electrophoresis (SDS-PAGE) and Western blotting of SarkoSpin pellets, 10% of SarkoSpin pellets per run were used for immunoblots. Samples were resuspended in final 1X LDS loading buffer (Invitrogen, NP0007) with 1X final Bolt sample reducing agent (Invitrogen, B0009), denatured at 70°C for 10 min, and loaded on Bolt 4-12% Bis-Tris gels (Invitrogen, NW04122BOX) using Bolt MES running buffer (Invitrogen, B0002) and Bolt Antioxidant (Invitrogen, BT0005) and run at 80V for 10 min and then at 115V. Gels were equilibrated for 10 min in 20% EtOH at room temperature and then transferred onto nitrocellulose membranes using iBlot 2 Transfer NC Stacks (Invitrogen, IB23001) with iBlot 2 Dry Blotting System (Invitrogen, IB21001) using a custom transfer method (23V for 8 min). Membranes were then blocked with 5% w/v non-fat skimmed powder milk in 0.05% v/v Tween-20 (Sigma-Aldrich, P1379) in PBS (milk PBST) and probed with primary antibodies (table S3) overnight at 4°C in PBST with 1% w/v milk, washed three times with PBST, followed by 1 hour incubation with HRP-conjugated secondaries in 1% milk PBST at room temperature. After three washes in PBST, immunoreactivity was visualized by chemiluminescence using SuperSignal West Pico or Femto Chemiluminescent Substrate (Thermo Scientific 34077, 34096) on Fusion FX6 EDGE imager.

### Immunoprecipitation

#### In vitro translation in HEK293T cell and RRL extracts:

RAN translation reactions were performed as previously described in HEK293FT cell-free translation extracts for 1 hour at 30°C or RRL extracts (26). The translation products were further incubated in a buffer containing 20 mM HEPES KOH pH 7.6, 100 mM KCl, 10% glycerol, 1 mM EDTA supplemented with 1 μg of N-ter (GA) antibody, an antibody raised against the N-terminal peptide of the GA frame (sequence MELRSRAL) overnight at 4°C or 1 μg of anti-HA antibody (table S3). Then either 50 μl of MagnaBind protein-A magnetic beads (Thermo Fisher Scientific; 21348) or a mix of 50% protein-A and 50% protein-G beads (Thermo Fisher Scientific; 21349) were added and incubated 4 hours on ice. Using a magnetic stand, the flowthrough was collected and the proteins bound on the beads were eluted by boiling the beads in protein loading buffer. The input, flowthrough and eluted fractions were further analyzed by Western blotting with antibodies directed against the three HA, His, and FLAG tags (table S3) as previously described (26).

#### HEK293T cells overexpressing G_4_C_2_ constructs:

The constructs used to transfect (Lipofectamine 2000, Invitrogen, 11668030) in HEK293T cells were described previously (26), and immunoprecipitation and Western blot were performed as previously described with minor changes (99). (99). Briefly, RIPA buffer (50 mM Tris HCl pH 8, 150 mM NaCl, 1% NP-40, 0.5% sodium deoxycholate, 0.1% SDS) with protease inhibitor (Roche, 4693116001) was used to lyse the cells. After measuring the protein concentration with BCA kit (Thermo Fisher Scientific, 23250), protein lysates were incubated with 1 μg of IgG antibody (Santa Cruz, sc-2027 and sc-2026) and 20 μl of Protein A/G beads (Santa Cruz, sc-2003) for 1 hour at 4°C on a rotor. Then, primary antibodies (table S3) were added to 1 mg of protein lysates and incubated at 4°C overnight on rotor. Next day, 35 μl of Protein A/G beads (Santa Cruz, sc-2003) were added to the protein lysates and incubated for 1 hour at 4°C on rotor. Then, the beads were spined down and washed four times with RIPA buffer (15 min each). Beads were spined down, 1X loading buffer (BIO-RAD, 1610737) was added and the samples were boiled at 100°C for 10 min and spined down again before loading.

Protein samples were loaded on 4 to 20% Precast Protein Gels (BIO-RAD, 5671093) and ran with running buffer (BIO-RAD, 1610772) at 120 V. Gels were equilibrated for 10 min in 20% EtOH at room temperature and then transferred onto PVDF membranes using iBlot 2 Transfer Stacks (Invitrogen, IB24001) with iBlot 2 Dry Blotting System (Invitrogen, IB21001) using a custom transfer method (23 V for 6 min). Membranes were then blocked with 5% w/v non-fat skimmed powder milk in 0.1% v/v Tween-20 (Sigma-Aldrich, P1379) in TBS and probed with primary antibodies (table S3) overnight at 4°C in TBST with 5% w/v bovine serum albumin (Sigma-Aldrich, A7906-500g). The next day, membranes were washed three times with TBST, followed by 2-hour incubation with HRP-conjugated secondaries (Abcam, AB205718 and AB97023) in 5% non-fat milk TBST at room temperature. After three washes in TBST, immunoreactivity was visualized by chemiluminescence using SuperSignal West Pico or Femto Chemiluminescent Substrate (Thermo Fisher Scientific 34077, 34096) on BIO-RAD ChemiDoc imager.

### Secondary structure probing

The CUG- and CCG-(G_4_C_2_)_66_ RNA transcripts containing 66 repeats were synthesized in vitro with T7 RNA polymerase as previously described (26). The RNA transcripts were then separated on denaturating PAGE containing 8 M urea and purified by electroelution using Bio Trap apparatus and Schleicher and Schuell membranes. The purified transcripts were then 32P-labeled by 5′ capping using the ScriptCap m7G Capping System kit from CELLSCRIPT. The radioactive RNA transcripts were probed directly after purification without any denaturation-renaturation step as previously described (100).

In-line probing (spontaneous cleavage probing) measures the intrinsic flexibility of an RNA molecule at each nucleotide. Flexible (usually unstructured or single-stranded) regions undergo spontaneous cleavage in the presence of Mg^2+^. In brief, 50,000 cpm of radiolabeled RNA was incubated in 50 mM Tris-HCl pH 8.8, 100 mM KCl without MgCl_2_ or with 1 or 10 mM MgCl_2_ for 72h at room temperature. The cuts in the RNA backbone were analyzed on denaturating PAGE containing 8 M urea. The band size and band intensity in the PAGE gel represents the sites and level of RNA flexibility.

RNase S1 probing is used to map the RNA single-stranded regions by selectively cleaving them with RNase S1. In brief, the RNA was incubated with serial dilutions of RNase S1 to have statistically one digestion cut per molecule for 10 min at room temperature as previously described (100). The cuts in the RNA backbone were analyzed on denaturating PAGE containing 8 M urea. The band size in the PAGE gel represents the sites of single-stranded RNA.

All the cuts from both probing methods were mapped by using an RNase T1 ladder performed in a denaturing buffer according to a previously established protocol (100). Indeed, RNase T1 cleaves after Guanine residues in single-stranded regions.

### Immunoassay

The protein lysates were prepared as previously described with minor modifications (30, 101). Briefly, frozen mouse cortex tissue was thawed on ice, homogenized in 5 × weight/volume of TE buffer [50 mM Tris (pH 7.4), 50 mM NaCl, 1 mM EDTA] with protease inhibitor cocktail (Roche, 4693116001) and phosphatase inhibitor cocktail (Sigma-Aldrich, P5726), and aliquoted. A 50 μl aliquot was thawed on ice and Benzonase buffer [12 mM MgCl2, 250U/sample Benzonase (Millipore, 71206)] was added to each sample, then incubated at room temperature for 5 min. 100 μl of coIP buffer [50 mM Tris-HCl (pH 7.4), 300 mM NaCl, 5 mM EDTA, 0.1% Triton X-100] with 3% SDS was added to each homogenate to achieve a final lysate with 2% SDS. For iPSC-derived neurons, 1 ml of RIPA buffer [150 mM NaCl, 50 mM Tris (pH = 8.0), 1% NP-40, 1% deoxycholate, 0.1% SDS] with protease inhibitor cocktail and phosphatase inhibitor cocktail were added per T75 flask of cells (~1 × 107 cells). For both brain and cell lysates, samples were sonicated in three cycles (Fisher Scientific, Sonic Dismembrator, Model 100, program 3), 1 to 5 s for each cycle and rested on ice between each cycle. BCA assay (Thermo Fisher Scientific, 23250) was used to measure the protein concentration.

Levels of DPR proteins in cell lysates were measured using the MSD electrochemiluminescence detection technology as previously described (102) with modifications, including an increase in the SDS concentration to 2% in the final lysate. 50 μg of protein lysate was used for poly-GA, 25 μg for poly-GP, and 125 μg for poly-GR in duplicate for the immunoassay in mouse tissue and doubled amounts of proteins were used for the measurements in iPSC-derived neurons. For capture, 96-well 1-spot SECTOR plates (MSD) were coated with the purified mouse monoclonal anti-GA antibody 5E9 (Millipore, MABN889) to detect poly-GA, or with the purified rat monoclonal anti-poly-GR antibody 5A2 (Millipore) to detect poly-GR. To detect poly-GP, a purified mouse monoclonal poly-GP antibody (TALS 828.179, Target ALS Foundation) was biotinylated and used to coat a 96-well MSD Small Spot Streptavidin plate. The same antibodies were conjugated to a SULFO-TAG NHS-Ester group to use as detection antibodies in each assay. For MSD combining N-ter (GA) and DPR antibodies, 96-well 1-spot SECTOR plates (MSD) were coated with the N-ter (GA) (customized purified rabbit polyclonal antibody, Rb9261) for capture. To detect poly-GA and poly-GP, purified mouse monoclonal anti-GA antibody 5E9 (Millipore, MABN889) and purified mouse monoclonal poly-GP antibody (TALS 828.179, Target ALS Foundation) were conjugated to a SULFO-TAG NHS-Ester group to use as detection antibodies in each assay.

### Cell culture

All cells were maintained at 37°C with 5% CO2 and routinely tested negative for mycoplasma.

#### HEK293T cells:

HEK293T cells were cultured in Dulbecco’s modified Eagle’s medium (DMEM) (Gibco, 11965092), supplemented with 10% fetal bovine serum (FBS) (Sigma-Aldrich, F0926) and antibiotics (Gibco, 15140122). Cells were transfected using Lipofectamine 2000 reagent (Invitrogen, 11668027). The total amount of plasmid DNA was adjusted to 20 μg per 10-cm dish. Cells were harvested at 48 hours after transfection for Western blot.

#### iPSCs and motor neuron differentiation:

iPSCs were obtained from skin fibroblasts of an ALS patient with a C9ORF72 expansion and a control as previously described (103). iPSCs were maintained in StemFlex medium (Thermo Fisher Scientific A3349401) on Vitronectin XF (STEMCELL, 07180) coated untreated plates.

Cells were differentiated into motor neurons using three independent methods: (i) piggyBac mediated expression of transcription factors (NGN2, ISL1, and LHX3: hNIL) (54, 56), (ii) a differentiation protocol based on small molecules (62, 63), or (iii) expression of the NGN2 transcription factor associated with small molecules (64).

The first differentiation method was performed as previously described (54, 56). Briefly, 90% confluent iPSCs were collected and nucleofected with equal concentrations of PiggyBac hNIL donor and PiggyBac transposase plasmids using the Amaxa Nucleofector II Device (Lonza) and the Nucleofector Human Stem Cell Kit (Lonza, VPH-5022). The hNIL construct is designed to allow the expression of the transcription factors NGN2, ISL1 and LHX3 in a Tet-ON system; it also includes a BFP reporter and a puromycin resistance gene for selection. The hNIL construct used here is a derivation of Addgene no. 197089 where we introduced the second intron of HBB into the NIL sequence to improve differentiation efficiency by reducing silencing of long cDNAs (104). Two days after nucleofection, medium was changed to iPSC medium + 2 μg/ml puromycin (InvivoGen, ant-pr-1) to allow selection of nucleofected cells. Nucleofected iPSCs were then expanded to confluence and then replated (day 0) in Vitronectin XF (Stemcell Technologies, 07180)-coated T75 flasks with induction medium [97% DMEM/F12 (Gibco, 11320033) + 1% N2 supplement (Gibco, 17502-048), 1% MEM Non-Essential Amino Acids Solution, NEEA (Gibco, 11140050), 1% GlutaMax (Gibco, 35050061), 2 μg/ml doxycycline (Sigma-Aldrich, D5207), and 0.1 μg/ml compound E (Millipore, 565790)]. After 3 days, cells were collected and replated in poly-L-ornithine (PLO, Sigma-Aldrich, P3655) coated plates (6 well-plates for protein and RNA collection, glass-bottom 96 well plates for imaging, day 3) with differentiation medium [Neurobasal medium (Gibco, 21103-049) + 1% N2 supplement (Gibco, 17502-048), 1% NEEA (Gibco, 11140050), 1% GlutaMax (Gibco, 35050061), 0.2% 2-Mercaptoethanol (Gibco, 31350010), 1 ng/ml BDNF (Life Technologies, PHC7074), 1 ng/ml GDNF (Stemcell Technologies, 78139.1), 1 ng/ml IGF-1 (R&D Systems, 291-G1-200), 1 ng/ml CNTF (R&D Systems, PHC7015), 10 nM retinoic acid (Sigma-Aldrich, R2625), 2 μg/ml doxycycline (Sigma-Aldrich, D5207), and 0.1 μg/ml compound E (Millipore, 565790)]. Half medium changes were performed every 3 days.

The small molecules–based differentiation method was performed as previously described (62, 63). Human iPSCs were differentiated into monolayer with mixed spinal neuron cultures. The differentiation includes three main stages. Stage 1 (day 0 to day 5) is for neuroepithelial induction. On day 0, iPSCs were dissociated and replated with 500,000 cells/well of 6-well plate and incubated with stage 1 medium: IMDM (Life Technologies, 12440053), F12 (Life Technologies, 11765062), NEAA (Life Technologies, 11140-050), B27 (Invitrogen, 17504044), N2 supplement (Gibco, 17502-048), Penicillin-Streptomycin (Invitrogen, 15140-122), LDN193189 (Stemgent, 04-0074-02), SB431542 (Stemcell Technologies, 72234), and CHIR99021 (Sigma-Aldrich, SML1046) with Rock Inhibitor (Y-27632, Sigma-Aldrich, 688000). Stage 1 medium was changed on day 1, day 3, and day 5. Stage 2 (day 6 to day 11) is for spinal neuron precursor generation. Cells were dissociated and replated with 2 × 106 cells/T25 flask and incubated with stage 2 medium: IMDM (Life Technologies, 12440053), F12 (Life Technologies, 11765062), NEAA (Life Technologies, 11140-050), B27 (Invitrogen, 17504044), N2 supplement (Gibco, 17502-048), Penicillin-Streptomycin (Invitrogen, 15140-122), LDN193189 (Stemgent, 04-0074-02), SB431542 (Stemcell Technologies, 72234), CHIR99021 (Sigma-Aldrich, SML1046), All-trans RA (Sigma-Aldrich, R2625), and SAG (Cayman Chemicals, 11914) with Rock Inhibitor (Y-27632, Sigma-Aldrich, 688000). Stage 2 medium was changed on day 7, day 9, and day 11. Stage 3 (from day 12) is for terminal spinal neuron maturation. Cells were dissociated and replated with 2 × 106 cells/well in a 6-well plate and incubated with stage 3 medium: IMDM (Life Technologies, 12440053), F12 (Life Technologies, 11765062), NEAA (Life Technologies, 11140-050), B27 (Invitrogen, 17504044), N2 supplement (Gibco, 17502-048), Penicillin-Streptomycin (Invitrogen, 15140-122), Compound E (Millipore, 565790), DAPT (Stemcell Technologies, 72082), db-cAMP (Millipore, 28745), All-trans RA (Sigma-Aldrich, R2625), SAG (Cayman Chemicals, 11914), Ascorbic Acid (Sigma-Aldrich, A4544), BDNF (Invitrogen, PHC7074), and GDNF (Stemcell technologies, 78139.1) with Rock Inhibitor (Y-27632, Sigma-Aldrich, 688000). Stage 3 medium was changed on day 13, and then twice per week until cell harvesting.

The transcription factor + small molecules protocol was performed as previously described with minor modification (64). From days 1 to 3, NGN2-nucleofected neurons were cultured in N2 medium supplemented with doxycycline (Sigma-Aldrich, D5207) and the following small molecules: LDN193189 (Stemgent, 04-0074-02), SB431542 (Stemcell Technologies, 72234), all-trans retinoic acid (RA; Sigma-Aldrich, R2625), and SAG (Cayman Chemicals, 11914). On day 4, the medium was replaced with Neurobasal medium supplemented with B27 (NBM-B27), doxycycline, BDNF (Life Technologies, PHC7074), GDNF (Stemcell Technologies, 78139.1), CNTF (R&D Systems, PHC7015), all-trans RA, and SAG. On days 5 and 6, the medium was refreshed with NBM-B27 containing doxycycline, BDNF, GDNF, CNTF, all-trans RA, SAG, and FUDR (Sigma-Aldrich, F0503). On day 7, cells were replated onto PLO (Sigma-Aldrich, P3655)–coated plates and maintained in NBM-B27 supplemented with BDNF, CNTF, GDNF, and growth factor–reduced Matrigel (Corning, 354230). The same medium was refreshed every 2 to 3 days thereafter.

### Base editing

Base editing was performed as previously described (52). iPSCs were passaged onto Vitronectin XF (STEMCELL, 07180)–coated 12-well plates at a density of 50,000 cells/well. Medium was changed, and transfections were performed once cells reached 70% confluence. 900 ng pEF_ABEMax base editor, 300 ng pEF1-XMAS, 300 ng CUG sgRNA (table S4) and 300 ng XMAS sgRNA was transfected per well using 4 μl Lipofectamine Stem transfection reagent (Thermo Fisher Scientific, STEM0001) in OptiMEM medium (Gibco, 11058-021) (52). Medium was changed to StemFlex medium (Gibco, A3349401) 24 hours posttransfection. Cells were dissociated using Accutase (Gibco, A11105-01) 48 hours posttransfection and passed through a 40-μm filter. Single iPSCs were fluorescence-activated cell sorted (FACS) according to their fluorescent signal [mCherry and/or green fluorescent protein (GFP)] into 96-well Vitronectin XF coated plates in StemFlex supplemented with CloneR2 (Stemcell Technologies, 100-0691). Medium was changed 7 days post-sort with fresh StemFlex and then every other day. Cell growth was monitored by optic microscopy. Once a colony grew to occupy half of the well surface, it was passaged to a 24-well plate and then to two wells of 12-well plate (one for expansion, the other for sequencing). Genomic DNA was extracted from the candidate clones using QIAamp DNA kits (Qiagen, 56304), and PCR was performed with Phusion High-Fidelity DNA Polymerases (Thermo Scientific, F531L) to amplify the sequence that contains the edit site (primer pair information in table S4). Sanger sequencing was performed at the MGH DNA core to validate the genotype. Off-target sites were predicted from CCTop (105). Sanger sequencing was performed to determine whether possible off-target sites were mutated (primer pairs information in table S4). Validated clones were expanded and frozen in liquid nitrogen for future use.

### RNA extraction and real-time RT-qPCR

Total RNA from mouse brain tissue and iPSC-derived motor neurons was extracted from TRIzol lysates as manufacturer’s protocol (Invitrogen, 15596026). RNA was quantified using a Nanodrop micro-volume spectrophotometer (Thermo Fisher Scientific). First strand cDNA synthesis and oligo dT priming were performed using the High-Capacity cDNA Reverse Transcription Kit (Thermo Fisher Scientific, 4368814) per manufacturer instructions. The qPCR step was performed using the iTaq Universal SYBR Green Supermix (BIO-RAD, 1725121). ACTB, Rpl13a, and Map2 were used as endogenous control (table S4). Three technical replicates were assayed per biological sample and analyzed on a CFX Connect Real-Time PCR Detection System (Bio-Rad). Results were quantified using the ΔΔCt method (106). Primer information can be found in table S4.

### Genotyping for C9ORF72 expansion using repeat-primed PCR

Genotyping for the G_4_C_2_ expansion was performed by repeat-primed PCR as described elsewhere (4). In brief, genomic DNA were extracted from the candidate clones using QIAamp DNA kits (Qiagen, 56304), and 100 ~ 300 ng DNA was used for 30 μl PCR system with Roche FastStart PCR Master (Roche, 4710436001), primer mix (table S4), DMSO, 5x Q-Solution and 25 mM MgCl2 (Qiagen 210220), 7-deaza-GTP 5 mM (NEB, N-445S), and non DEPC-treated nuclease-free water (Invitrogen, AM9937). A touchdown PCR cycling program was used where the annealing temperature was gradually lowered from 70° to 56°C in 2°C increments with a 3-min extension time (at 72°C) for each cycle (4). PCR products underwent DNA fragment analysis at the MGH DNA Core and results were viewed with Peak Scanner Software 2.

### Immunocytochemistry, image acquisition, and analysis

iPSCs and iPSC-differentiated neurons in glass-bottom 96-well plate (Cellvis) were fixed for 15 min at room temperature with 4% paraformaldehyde and gentle washed three times with PBS. Fixed cells were treated with PBST (PBS with 0.2% Triton X-100) at 4°C for 10 min for membrane permeabilization. Blocking was done with 1% BSA in PBS then followed by primary antibodies incubation (in blocking buffer) overnight. The next day, the primary antibody was removed and the cells were gently washed three times with PBS then incubated with secondary antibodies (Alexa Fluor, Invitrogen, 1:500) at room temperature for 1 hour. Cells were washed three times with PBS. DAPI (in PBS, 15 min) was used to label the nuclei. Cells were kept in PBS before taking images. For imaging of iPSC markers and neuronal markers, a Nikon C2 confocal microscope was used. For STING quantification, confocal images were acquired with an Image X-Press Micro Confocal (Molecular Devices) or LSM900 confocal microscope (Zeiss) as described previously (43). Four z-stacks were acquired per field (1.6-μm step size), 15 to 25 fields per well were taken using a 40X objective. Automatic quantifications were performed using a custom Fiji/ImageJ-based plugin (National Institutes of Health, version 1.53c) (43).

### hNIL motor neurons survival assay

Because the PiggyBac hNIL plasmid includes a nuclear BFP2, successfully nucleofected cells displayed blue, fluorescent nuclei, allowing us to count them over time. hNIL motor neurons at day 3 of differentiation were seeded in a poly-L-ornithine (Sigma-Aldrich, P3655) coated glass-bottom 96-well plate (Cellvis) at a density of 50,000 cells/well. Day 3 medium contained aphidicolin (Cell Signaling, 32774), a DNA polymerase inhibitor, to eliminate actively proliferating cells. At day 4, cells underwent a full medium change, and a baseline count was performed. Counting was repeated on day 7, and day 11. For imaging, we used an ImageXpress MicroConfocal (Molecular Devices), to image the entire well (25 images with a 10X magnification) in both brightfield and blue fluorescence (450 nm) spectrum. Four wells per genotype were longitudinally analyzed. The number of motor neurons was quantified by counting the number of BFP^+^ nuclei using custom scripts (https://github.com/waingerlab/Jiang_etal) (107).

### RNA-seq and analysis

iPSC derived motor neurons were collected in Trizol, RNA extracted and shipped to Azenta Life Sciences (South Plainfield, NJ, USA). RNA samples were quantified using Qubit 2.0 Fluorometer (Life Technologies, Carlsbad, CA, USA) and RNA integrity was checked using Agilent TapeStation 4200 (Agilent Technologies, Palo Alto, CA, USA). ERCC RNA Spike-In Mix 1 (ThermoFisher Scientific, cat. no. 4456740) was added to normalized RNA samples before initiating library preparation following the manufacturer’s protocol. RNA-seq libraries were prepared using the NEBNext Ultra II RNA Library Prep Kit for Illumina and the NEBNext Poly(A) mRNA Magnetic Isolation Module following the manufacturer’s instructions (NEB, Ipswich, MA, USA). Briefly, mRNAs were initially enriched with Oligod(T) beads. Enriched mRNAs were fragmented for 15 min at 94°C. First-strand and second-strand cDNA were subsequently synthesized. cDNA fragments were end-repaired and adenylated at 3′ ends, and universal adapters were ligated to cDNA fragments, followed by index addition and library enrichment by PCR with limited cycles. The sequencing library was validated on the Agilent TapeStation (Agilent Technologies, Palo Alto, CA, USA), and quantified by using Qubit 2.0 Fluorometer (Invitrogen, Carlsbad, CA) as well as by qPCR (KAPA Biosystems, Wilmington, MA, USA). The sequencing libraries were clustered on a flowcell. After clustering, the flowcell was loaded on the Illumina NovaSeq X Plus instrument according to the manufacturer’s instructions. The samples were sequenced using a 2 × 150 bp Paired-End (PE) configuration, targeting ~30 million reads per sample. The control software conducted image analysis and base calling.

Raw sequence data (.bcl files) generated by the sequencer were con verted into fastq files and de-multiplexed using Illumina’s bcl2fastq 2.20 software. One mismatch was allowed for index sequence identification. The fastq sequencing files were used to align with human reference genome (hg38) using STAR (version 2.7.9a) (108). The raw counts were subjected to differential gene expression using the DESeq2 package in R (version 4.2.1) (109). Gene expression was normalized with transcripts per million (TPM) values for each gene. Up- and down-regulated genes were all genes with a *P_adjust_* < 0.05 and a positive or negative log2fc. Volcano plots were produced using DEseq data with an additional filter of expressed genes as described previously (110) and “lfcShrink” ashr function (111). GO analysis was conducted using ShinyGO 0.77 (112). Background genes were set as genes tested for differential expression (all genes with a p-adjusted value not equal to “NA” and that passed the TPM cut-off determined from bimodal distribution of gene expression).

### PI uptake

PI uptake assays were conducted using neuronal cultures stained with PI (Biotium, no. 40017) at a final concentration of 0.5 μg/ml as previously reported (67). PI was diluted in complete growth medium and applied to the cells concurrently with toxin treatment. Imaging and analysis were performed using the IncuCyte S3 Live-Cell Analysis System and its accompanying software (Sartorius, version 2018B). For time course experiments, images were acquired every 12 hours post-treatment using the IncuCyte ZOOM Plan Fluor 20×/0.45 objective lens (Sartorius, cat. no. 4465). Neurons positive for PI were defined as those exhibiting fluorescence intensity at least 0.5 red calibrated units (RCU) above background, determined after Top-Hat background subtraction with a 10 μm radius. To establish the maximum number of cells per field of view, 10% Triton X-100 was added at the conclusion of each experiment to induce complete cell lysis, and PI-positive cells were quantified. The percentage of PI uptake at each time point was calculated relative to this maximal count. For the data presented, baseline PI counts were subtracted from all subsequent time points.

### TUJ1 immunocytochemistry and analysis

TUJ1 staining was done as described (66, 67). Briefly, cells were fixed with 4% paraformaldehyde and 15% sucrose in PBS for 1 hour at room temperature. The cells were then permeabilized with 0.3% Triton-X-100 in PBS for 1 hour at room temperature and blocked with 1% BSA in PBS for 1 hour at room temperature. Fluorescently conjugated 647-TUJ1 antibody (Biolegend cat. no. 801210) at a dilution of 1:1000 and 488-NeuN antibody (Biolegend cat. no. 608456) at a dilution of 1:500 was applied to the cells in 1% BSA + 0.3% Triton-X-100 in PBS overnight at 4C. Cells were then rinsed 3x in PBS and imaged using a Leica Thunder microscope with Andor Zyla sCMOS camera via a 20× Plan Apo objective.

The microtubule (MT) depolymerization index was measured using a custom macro adapted from a previous study (66). Briefly, polymerized microtubules were distinguished from depolymerized microtubules based on differences in brightness and circularity. Polymerized microtubules exhibit lower brightness and reduced circularity compared with the more aggregated and circular depolymerized tubulin structures. Using a custom analysis macro, an initial low-intensity threshold is applied to identify all TUJ1-positive structures, encompassing both polymerized and depolymerized microtubules. A subsequent high-intensity threshold is then applied to selectively detect the brighter regions corresponding to depolymerized microtubules. Once identified and masked, the pixels corresponding to depolymerized microtubules are subtracted from the initial mask to isolate the pixel set representing polymerized microtubules alone. These masks are further refined by applying a circularity filter to enhance specificity. The final mask for polymerized microtubules is then skeletonized to facilitate downstream quantification.

Microtubule (MT) depolymerization was quantified using a depolymerization index, defined as:

MT Depolymerization Index=Area of depolymerized microtubules/(Area of depolymerized microtubules+Area of polymerized microtubules).

To assess neurite density, NeuN-positive nuclei were detected and quantified using simple thresholding techniques. Neurite density was calculated as the total length of polymerized microtubules normalized to the number of NeuN-positive nuclei.

All threshold parameters used in the microtubule depolymerization analysis were empirically determined using positive and negative control samples, before the analysis of the complete datasets.

### Super-resolution STED microscopy and analysis

Cells were fixed with 4% paraformaldehyde for 15 min then washed three times with PBS. Permeabilization was performed using 0.1% (w/v) Triton X-100 (Thermo Scientific, cat. no. 85111) in PBS for 10 min at room temperature, followed by three additional PBS washes. Cells were then blocked in 3% (w/v) BSA (Sigma-Aldrich, cat. no. 2930) in PBS for 1 hour at room temperature. Primary antibodies diluted in 3% BSA were incubated with the cells overnight at 4°C. After three washes with PBST [PBS containing 0.1% (v/v) Tween-20], cells were incubated with secondary antibodies, also diluted in 3% BSA, for 1 hour at room temperature. The secondary antibodies used were: abberior STAR RED goat anti-mouse IgG (STRED-1001-500UG, 1:500) and Alexa Fluor 488-conjugated anti-chicken (1:2000). After incubation, cells were washed three times with PBST and counterstained with 100 ng/ml DAPI in PBS for 3 min at room temperature. Finally, cells were washed three more times with PBS. Primary antibodies information can be found in the table S3.

STED imaging was performed using an Abberior Instruments Expert Line system (Abberior) implemented on a Nikon Eclipse TE2000-U inverted microscope (Nikon) equipped with a 100× oil immersion objective (NA 1.45; Nikon, MRD71970). For imaging Mab414 labeled with Abberior STAR RED, excitation was performed with a 640-nm laser, depletion was achieved using a 775-nm STED laser, and fluorescence was detected at 660 nm. Map2, labeled with Alexa Fluor 488, was excited with a 488-nm laser and detected at 520 nm without STED depletion. DAPI was imaged using 405-nm excitation and detected at 461 nm, without STED depletion.

Mab414 foci were detected using the “Find Maxima” function in Fiji. To quantify mislocalized Mab414 foci, CellProfiler was used. Nuclear regions were defined based on DAPI signals using the “MedianFilter” and “IdentifyPrimaryObjects” modules. To measure the number of Mab414 foci mislocalized within the nucleus, the “ExpandOrShrinkObjects” module was applied to define an annular region located 700 to 1200 nm from the nuclear boundary. The number of foci within this region was then normalized by the total number of Mab414 foci inside the nucleus to account for variations in nuclear size.

## Supplementary Material

Jiang et al Supplementary Material

Supplementary Table 2

Supplementary Table 3

Supplementary Table 4

Supplementary Table 1

## Data Availability

All data are available in the main text or the supplementary materials. RNA-seq data generated in this manuscript have been deposited in dbGaP (phs002440.v3.p1). These data are available under restricted access to comply with the conditions of the informed consent form agreed with the donors involved in the study, and access can be obtained by applying for controlled access through the dbGaP website. Antibodies against C9ORF72 dipeptide repeat proteins are available from F.-B.G. and C.L.-T. under a material transfer agreement with the University of Massachusetts and Massachusetts General Hospital, respectively.
