# Supplementary material for: Blocking RAN translation without altering repeat RNAs rescues C9ORF72-related ALS and FTD phenotypes
Source: Science. Author manuscript; Available in PMC 2026 Apr 24. (PMC13107528; doi:10.1126/science.adv2600)
Supplement: Jiang et al Supplementary Material [file NIHMS2161796-supplement-Jiang_et_al_Supplementary_Material.docx]

Supplementary Materials for

**Blocking RAN translation without altering repeat RNAs rescues *C9ORF72*-related ALS/FTD phenotypes**

Xin Jiang, Laure Schaeffer, Divya Patni, Tommaso Russo, Chao-Zong Lee, Corey Aguilar, Christine Marques, Karen Jansen-West, Marian Hruska-Plochan, Ananya Ray-Soni, Su Min Lim, Aaron Held, Mei Yue, Paula Castellanos Otero, Sandeep Aryal, Hortense D. A. M. Beaussant, Himanish Basu, Hiro Takakuwa, Lillian M. Daughrity, Nandini Ramesh, Paulo Da Costa, Ana Rita A. A. Quadros, Matthew Nolan, Charles Jourdan F. Reyes, Hayden Wheeler, Laura C. Moran, Grant Griesman, Benjamin Wymann, Bianca A. Trombetta, Emma Sofia Lopez-De-Silanes, Michael Canori, Gopinath Krishnan, Yasmim Vieira Souza Da Silva, Gilbert Eriani, Mark W. Albers, Steven E. Arnold, Yuyu Song, Ankur Jain, Isaac M. Chiu, Yong-Jie Zhang, Fen-Biao Gao, Brian J. Wainger, Magdalini Polymenidou, Leonard Petrucelli, Franck Martin, Clotilde Lagier-Tourenne

Correspondence to: Franck Martin (email: [f.martin@ibmc-cnrs.unistra.fr](mailto:f.martin@ibmc-cnrs.unistra.fr)), Clotilde Lagier-Tourenne (email: [clagier-tourenne@mgh.harvard.edu](mailto:clagier-tourenne@mgh.harvard.edu))

**
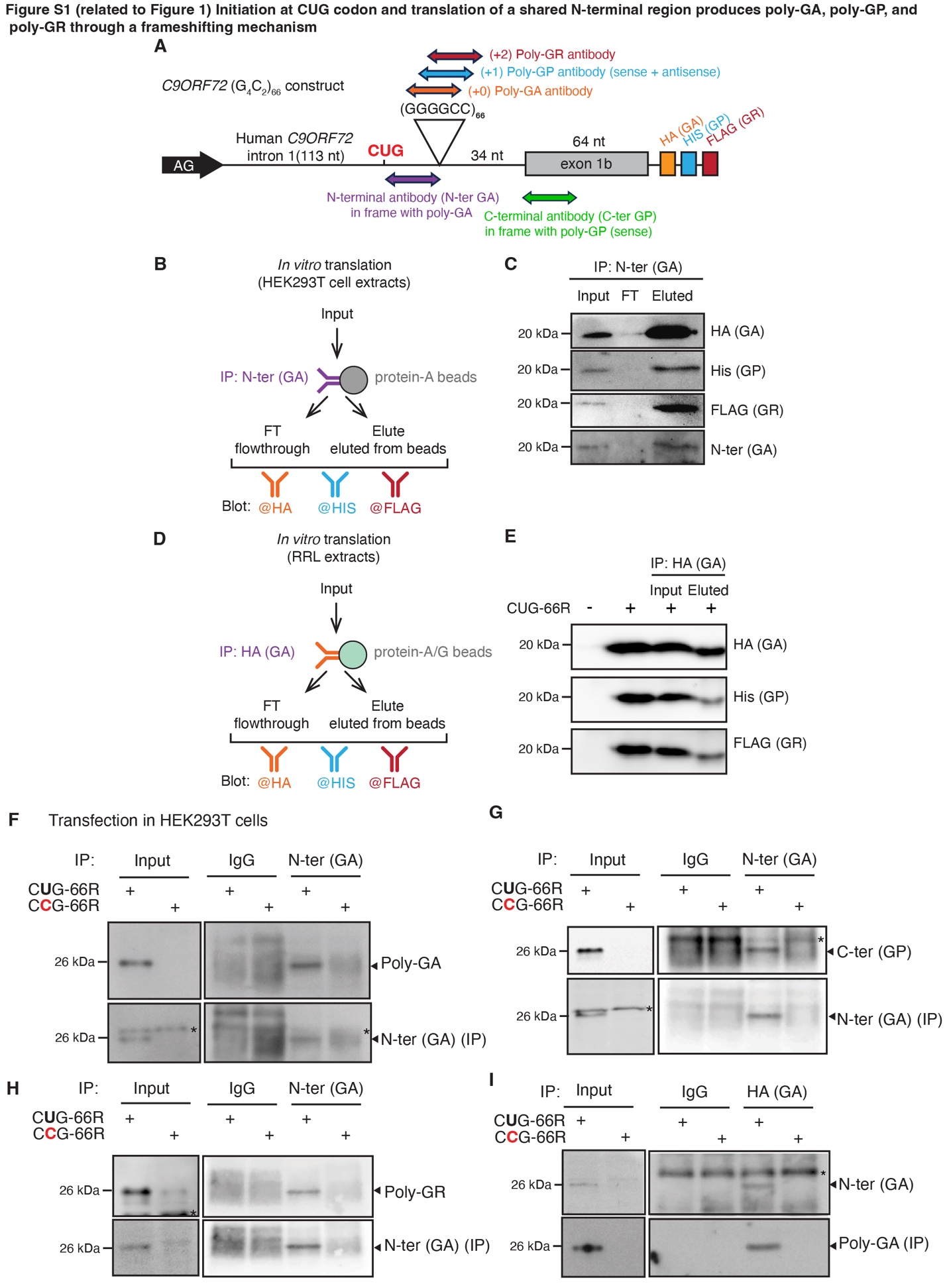
Figure S1**

**Fig. S1. Initiation at CUG codon and translation of a shared N-terminal region produces poly-GA, poly-GP, and poly-GR.** (**A**) Schematic of the transgene and antibodies used in this study. The repeat expansion is driven by an AG promoter (CMV enhanced chicken beta-actin) and flanked by sequences of human *C9ORF72* gene. Three tags (HA, 6xHIS, and FLAG) are inserted in the reading frames of poly-GA, poly-GP, and poly-GR, respectively. Antibodies targeting poly-GA, poly-GP (sense and antisense), poly-GR, C-terminal of poly-GP (sense), and N-terminal of poly-GA were used in this study. (**B**) Schematic of DPRs immunoprecipitation with an N-terminal antibody in frame with poly-GA (N-ter GA) and immunoblotting with antibodies against HA, HIS and FLAG translated from each of the three frames, poly-GA, poly-GP and poly-GR, respectively. Immunoprecipitation was performed after *in vitro* translation of CUG-(G_4_C_2_)_66_ RNA in HEK293T cell lysates. (**C**) Western blot with HA, HIS or FLAG antibodies of the input, flowthrough (FT) or eluted proteins from protein-A beads after immunoprecipitation with the N-ter (GA) antibody. (**D**) Schematic of DPRs immunoprecipitation with an HA antibody and immunoblotting with antibodies against HA, HIS and FLAG translated from each of the three frames, poly-GA, poly-GP and poly-GR, respectively. Immunoprecipitation was performed after *in vitro* translation of CUG-(G_4_C_2_)_66_ RNA in Rabbit Reticulocyte Lysate (RRL) lysates. (**E**) Western blot with HA, HIS or FLAG antibodies of the input and eluted proteins from protein-A/G beads after immunoprecipitation with the HA antibody. The first lane is a negative control with only RRL, the second lane is RRL with CUG-66R RNA to show the location of DPRs. (**F-I**) CUG-(G_4_C_2_)_66_ construct was transfected into HEK293T cells, and immunoprecipitation with N-ter (GA) antibody (**F, G, H**), HA (GA) antibody (**I**) or IgG control was performed using RIPA buffer. Immunoblotting of the input or eluate after immunoprecipitation was performed using poly-GA (**F**), C-ter (GP) (**G**), poly-GR (**H**), and N-ter (GA) (**I**), separately.

**
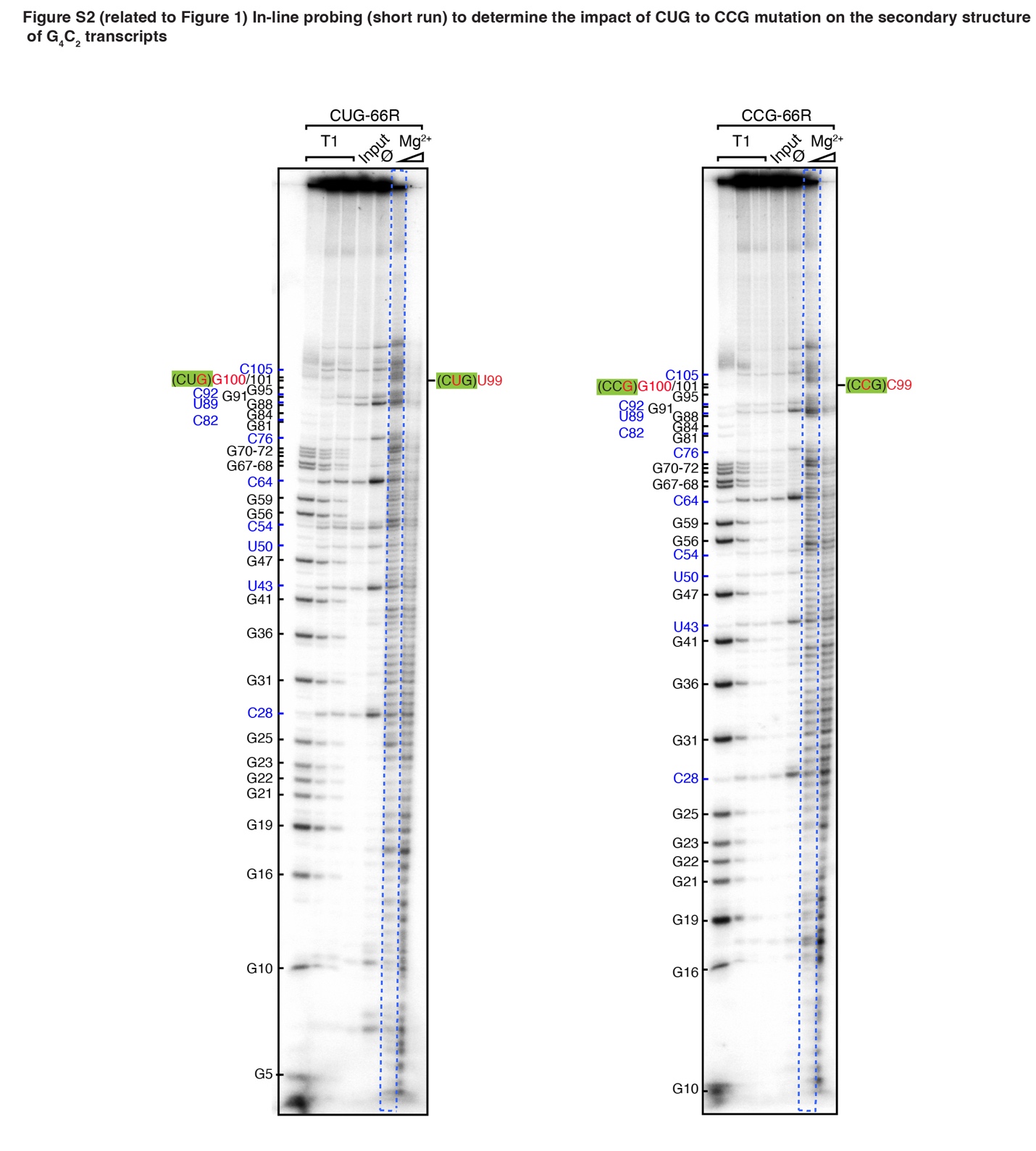
Figure S2**

**Fig. S2. In-line probing (short run) to determine the impact of CUG to CCG mutation on the secondary structure of G_4_C_2_ transcripts.** In-line probing of 5’radiolabelled CUG- (left panel) and CCG-(G_4_C_2_)_66_ (right panel) transcripts. The short migration enables the probing of the 5’ end of each transcript. The RNase T1 ladder is shown on the left of each panel. The position of the G residues (which are cut by the RNase T1) is indicated in black, while spontaneous cuts occurring at UA or CA are indicated in blue. The G of the CUG codon is shown in red. The mutation U to C at position 99 is highlighted in green. Input/*⏀* is the RNA not exposed to Mg^2+^. The reactivity pattern in the presence of 10 and 100 mM Mg^2+^ is shown in the last two lanes on the right of each panel where the bands represent the sites of flexible/unstructured RNA. The reactivity pattern with Mg^2+^ (highlighted in blue dash frame) showed similar RNA structures between the CUG and CCG-containing transcripts.

**
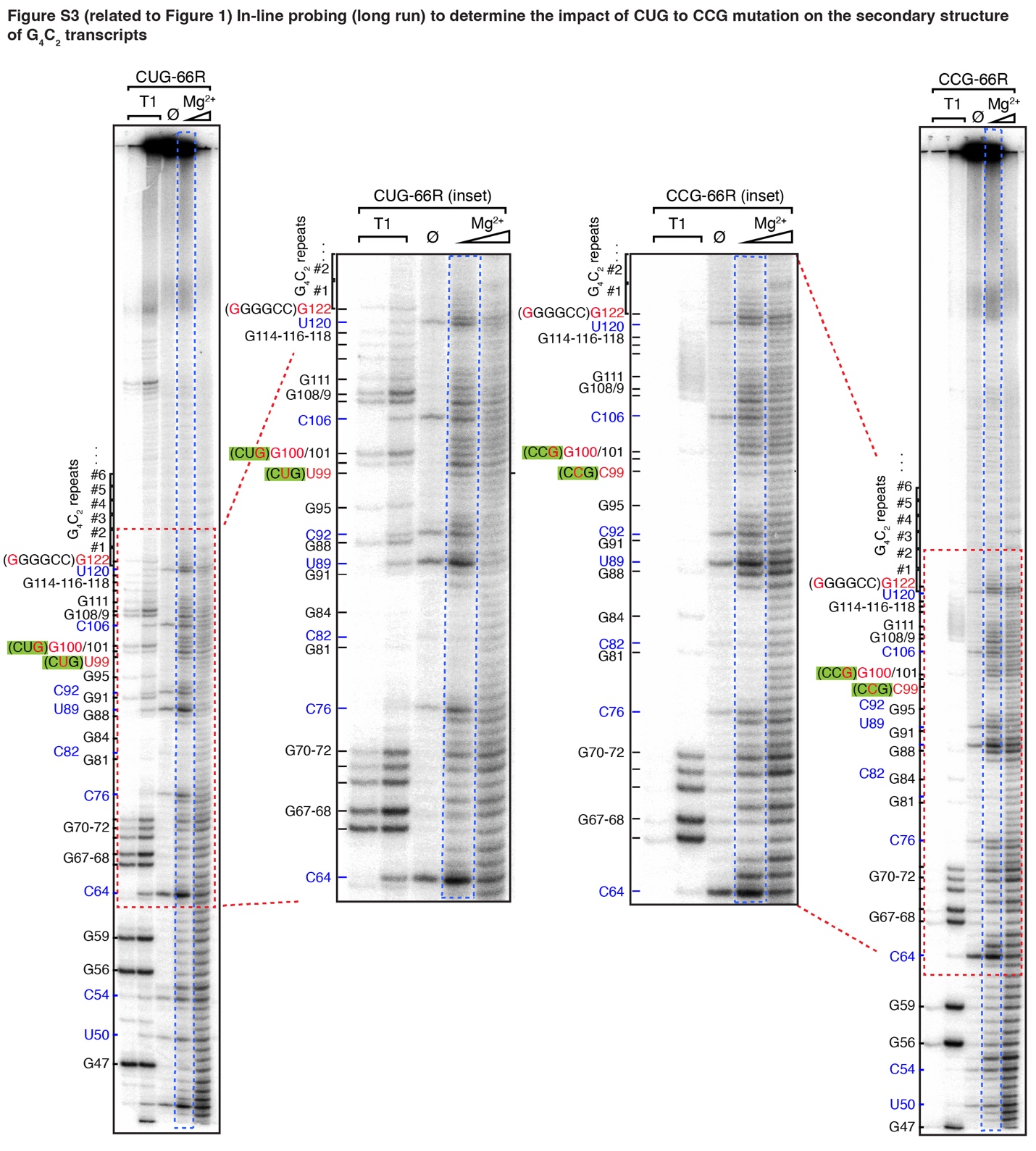
Figure S3**

**Fig. S3.** **In-line probing (long run) to determine the impact of CUG to CCG mutation on the secondary structure of G_4_C_2_ transcripts.** In-line probing of 5’radiolabelled CUG- (left panel) and CCG-(G_4_C_2_)_66_ (right panel) transcripts with zoom-in windows focus on the region of the CUG codon. The long migration enables the probing of the region of the CUG codon and the beginning of the repeats. The RNase T1 ladder is shown on the left of each panel. The position of the G residues is indicated in black, spontaneous cuts occurring at UA or CA are indicated in blue. The G of the CUG codon is shown in red. The first G of the first repeat is also shown in red. The mutation U to C at position 99 is highlighted in green. The position of the first repeats is indicated on the right of each panel. *⏀* is the RNA not exposed to Mg^2+^. The reactivity pattern in the presence of 10 and 100 mM Mg^2+^ is shown in the last two lanes on the right of each panel where the bands represent the sites of flexible/unstructured RNA. The reactivity pattern with Mg^2+^ (highlighted in blue dash frame) showed similar RNA structures between the CUG and CCG-containing transcripts.

**
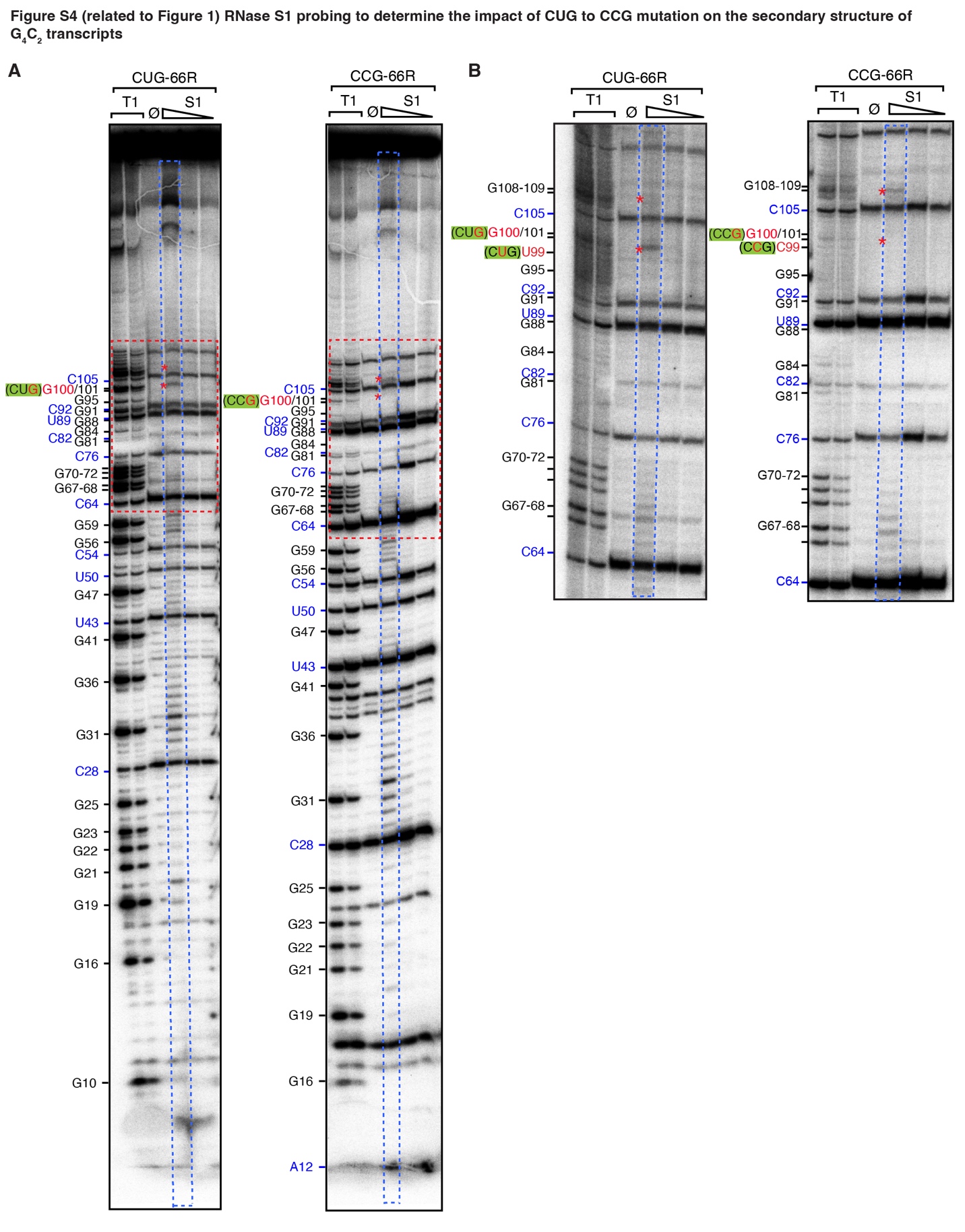
Figure S4**

**Fig. S4.** **RNase S1 probing to determine the impact of CUG to CCG mutation on the secondary structure of G_4_C_2_ transcripts.** (A-B) RNase S1 probing of 5’radiolabelled CUG- (left panels) and CCG-(G_4_C_2_)_66_ (right panels) transcripts. The short migration (A) enables the probing of the 5’ end of each transcript. The long migration (B) enables a focus on the region of the CUG codon. The RNase T1 ladder is shown on the left of each panel. The position of the G residues is indicated in black, spontaneous cuts occurring at UA or CA are indicated in blue. The G of the CUG codon is shown in red, with CUG/CCG highlighted in green. The mutation U to C at position 99 is highlighted in red. *⏀* is the RNA not exposed to RNase S1. The RNase S1 cleavage pattern in the presence of increasing concentration of RNase S1 is shown on the last three lanes on the right of each panel where the bands represent the sites of single-stranded RNA. The reactivity pattern with the highest concentration of RNase S1 (highlighted in blue dash frame) showed highly similar RNA structures between the CUG and CCG-containing transcripts. Two minor changes in the vicinity of the CUG and CCG mutant are marked by red *.

**
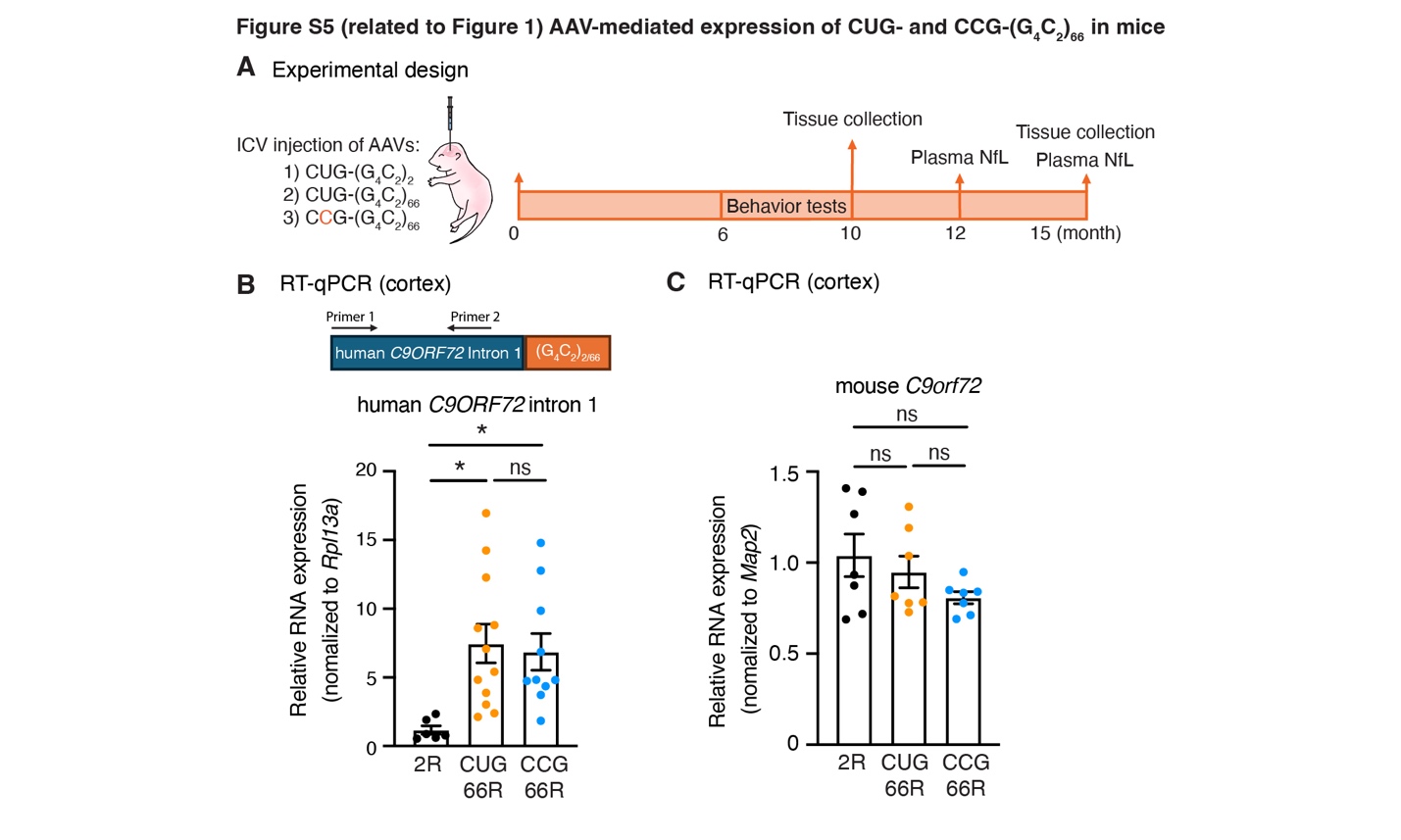
Figure S5**

**Fig. S5. AAV-mediated expression of CUG- and CCG-(G_4_C_2_)_66_ in mice.** (**A**) Experimental design for *in vivo* evaluation of the DPRs contribution on pathological and behavioral phenotypes in mice injected with AAV to express (G_4_C_2_)_2_ or (G_4_C_2_)_66_ with either a CUG or mutated CCG codon upstream of the repeat. (**B**) RT-qPCR determining the levels of the transgene using primers in the human *C9ORF72* intron 1b in cortex of 10-month-old AAV-injected mice. N = 6-12 mice per genotype, each dot represents the value from one animal. Mean ± SEM; one-way ANOVA with Tukey’s multiple comparisons test; * P<0.05. (**C**) RT-qPCR determining the levels of the mouse *C9orf72* in cortex of 10-month-old AAV-injected mice. N = 7 mice per genotype, each dot represents the value from one animal. Mean ± SEM; one-way ANOVA with Tukey’s multiple comparisons test.

**
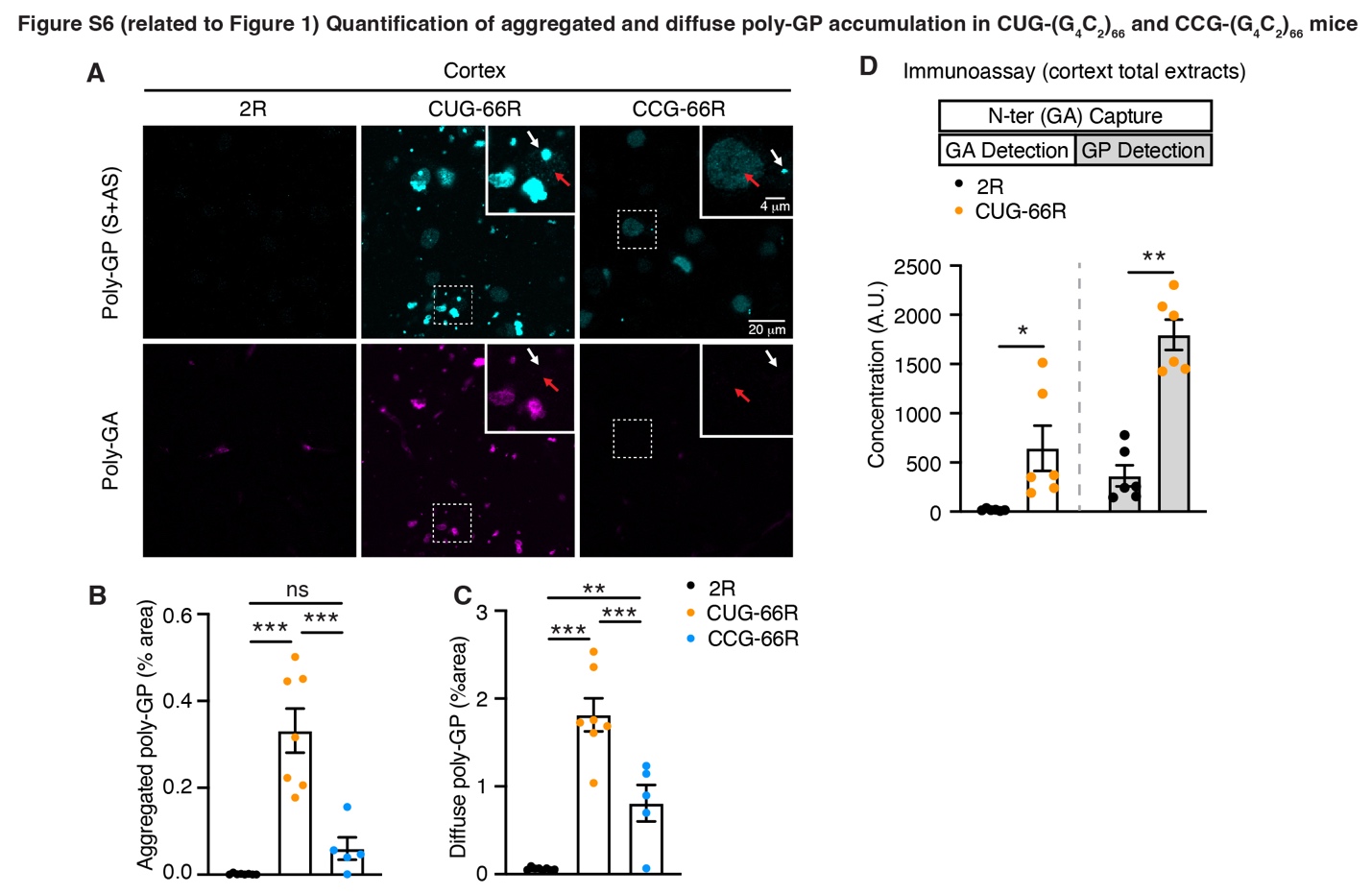
Figure S6**

**Fig. S6. Quantification of aggregated and diffuse poly-GP accumulation in CUG-(G_4_C_2_)_66_ and CCG-(G_4_C_2_)_66_** **mice.** (**A**) Co-immunostaining of poly-GP with poly-GA in cortex of AAV-injected 15-month-old mice. White arrow heads indicate aggregated forms of poly-GP which are not co-localized with poly-GA. Red arrow heads indicate diffused forms of poly-GP which are also not overlapping with poly-GA staining. (**B, C**) The percentage area covered by aggregated (**B**) and diffuse (**C**) poly-GP was quantified. N=5-7 mice per genotype, each dot represents the average value from two brain sections from the same mouse. Mean ± SEM; one-way ANOVA with Tukey’s multiple comparisons test; ** P<0.01, *** P<0.001. (**D**) N-ter (GA)/GA and N-ter (GA)/GP MSD immunoassay was used to detect the levels of poly-GA and poly-GP in the mouse brain lysates containing 2% SDS with the N-ter (GA) as the capture antibody and GA/GP as the detection antibodies. N=6 technical replicates with 2 replicates/animal. Mean ± SEM; one-way ANOVA with Tukey’s multiple comparisons test; * P<0.05, ** P<0.01.

**
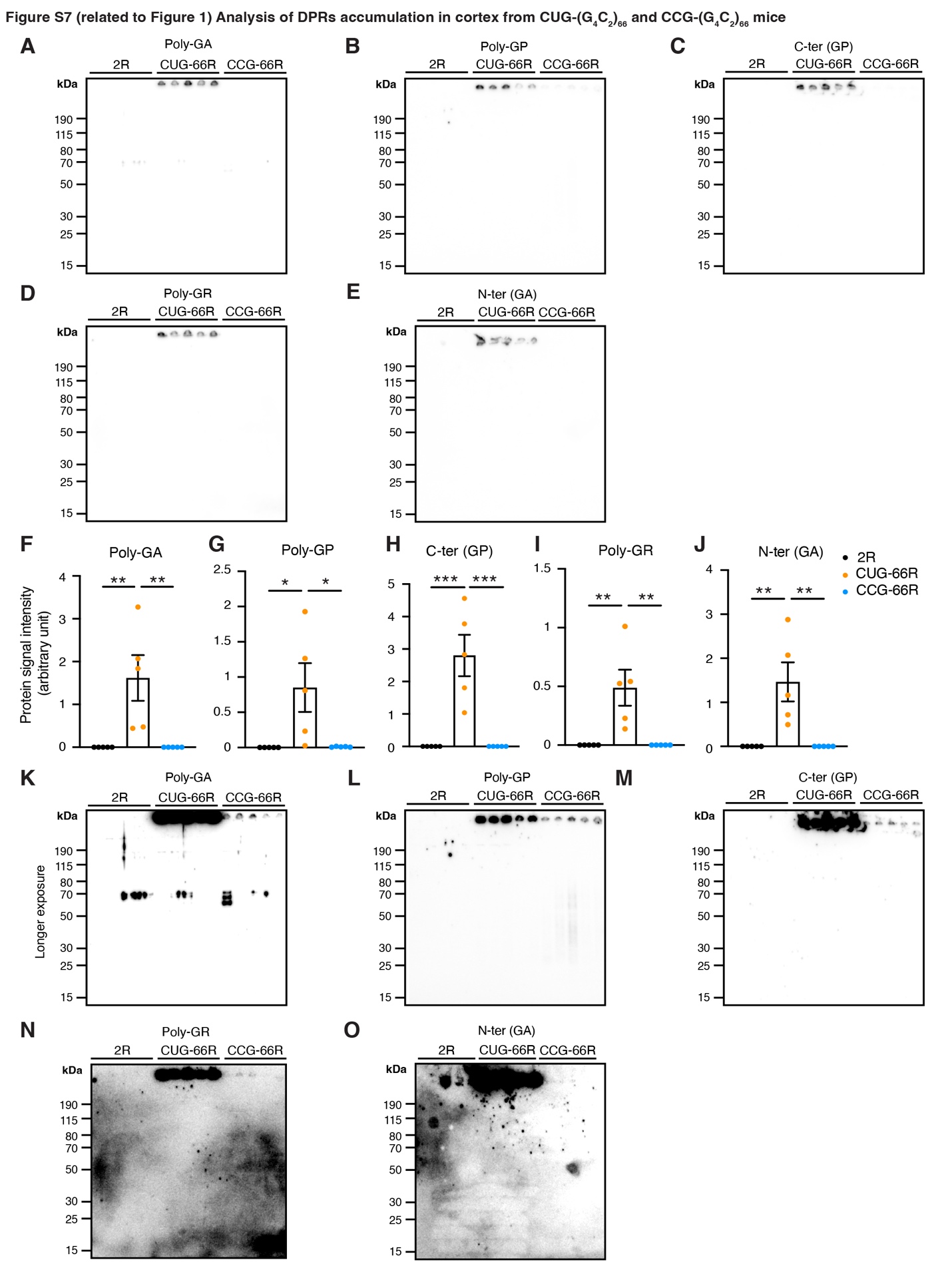
Figure S7**

**Fig. S7. Analysis of DPRs accumulation in cortex from CUG-(G_4_C_2_)_66_ and CCG-(G_4_C_2_)_66_** **mice.** (**A-J**) Full western blot images (cropped figures in Fig.1H) and quantification showing the expression of DPRs in SarkoSpin pellet fractions from cortex tissues of 15-month-old mice. N=5 mice per genotype. Mean ± SEM; one-way ANOVA with Tukey’s multiple comparisons test; * P<0.05, ** P<0.01, *** P<0.001. (**K-O**) Same blots as **A**-**E** but with longer exposure time.

**
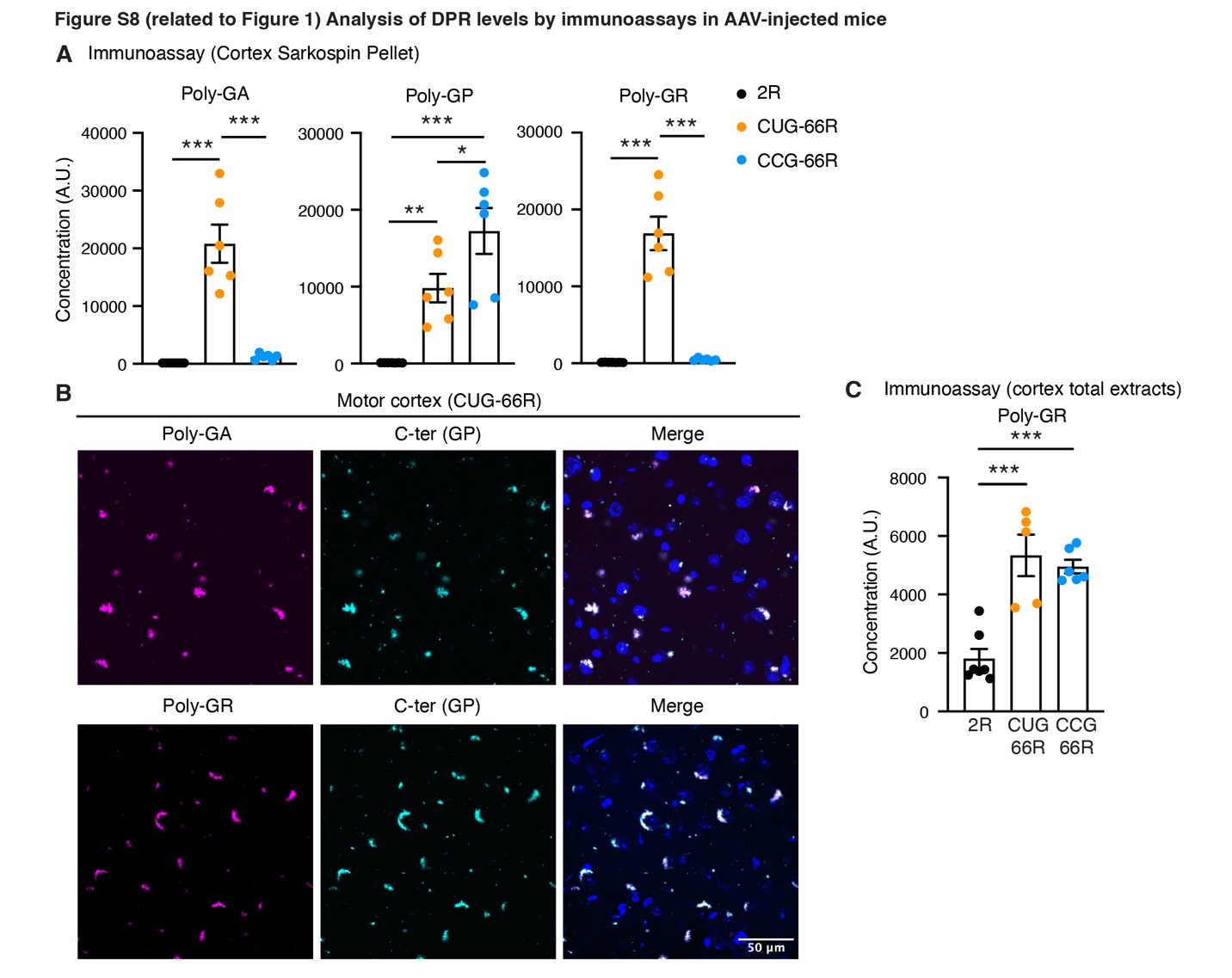
Figure S8**

**Fig. S8. Analysis of DPR levels by immunoassays** **in AAV-injected mice.** (**A**) Levels of poly-GA, poly-GP, and poly-GR in 15-month-old mouse cortex from SarkoSpin pellet fraction were measured using immunoassays. N=6 mice per genotype, mean ± SEM, two-way ANOVA with Tukey’s multiple comparisons test, * P<0.05, *** P<0.001. (**B**) Co-immunostaining of poly-GA with C-ter poly-GP (upper panels) and poly-GR with C-ter poly-GP (lower panels) in the cortex of 15-month-old AAV-(G_4_C_2_)_66_ mice. (**C**) Level of poly-GR measured with immunoassay in total extracts from cortex of 15-month-old mice. N=5-6 mice per genotype, mean ± SEM, one-way ANOVA with Tukey’s multiple comparisons test, *** P<0.001.

**
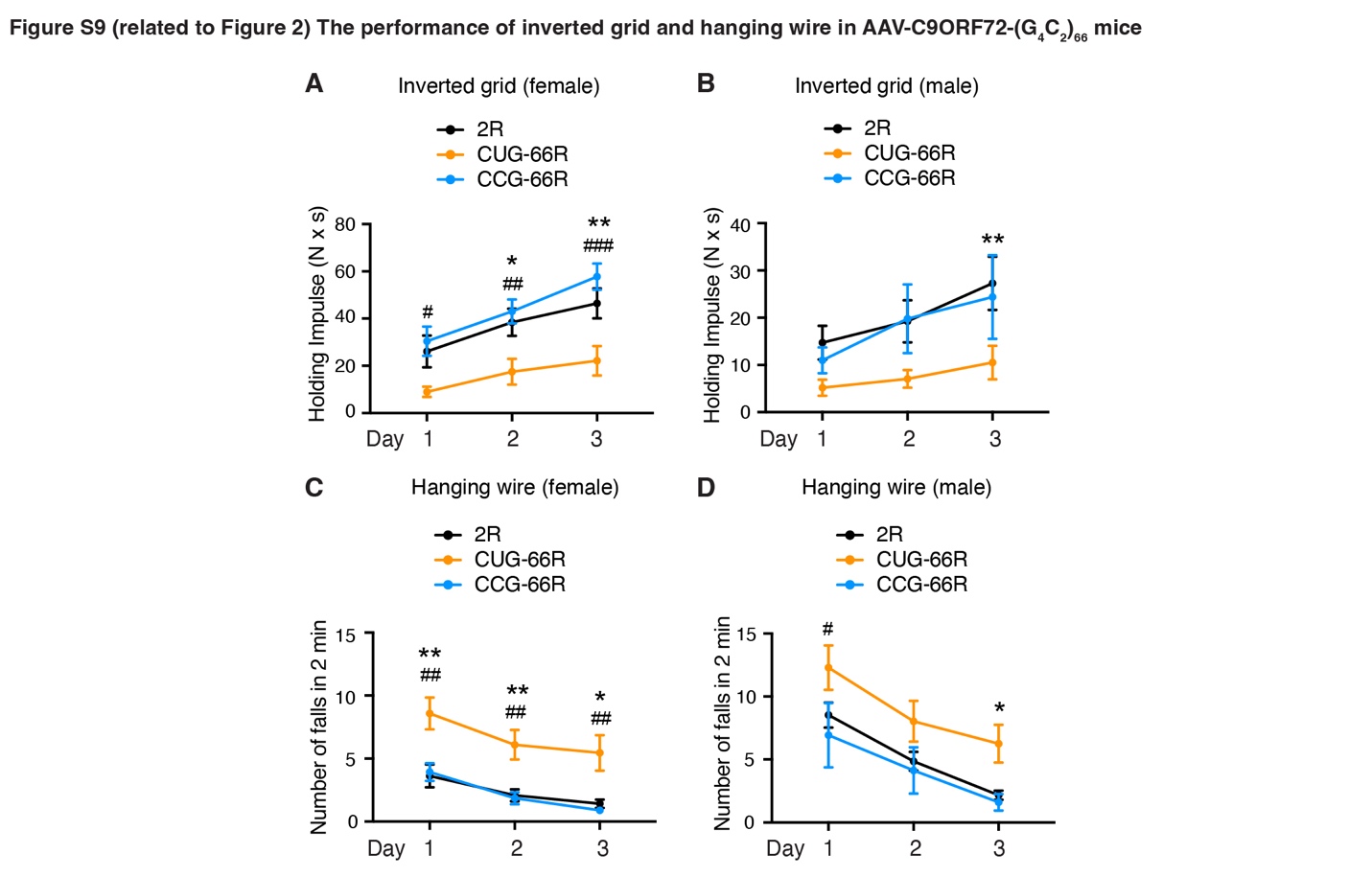
Figure S9**

**Fig. S9. The performance of inverted grid and hanging wire in AAV-*C9ORF72*-(G_4_C_2_)_66_ mice.** (**A, B**) Inverted grid test performed in 9-month-old female (**A**) and male (**B**) mice. Holding impulse = body weight (g) x 0.00980665 (N/g) x hanging time (s). N = 8-14 mice per genotype. (**C, D**) Hanging wire test determined the number of falls within 2 min in 9-month-old female (**C**) and male (**D**) mice. N = 5-12 mice per genotype, mean ± SEM, two-way ANOVA with Tukey’s multiple comparisons test, *: differences between 2R and CUG-149R; #: differences between CUG-149R and CCG-158R; ^: differences between 2R and CCG-158R. * P<0.05, ** P<0.01, *** P<0.001.

**Figure S10**


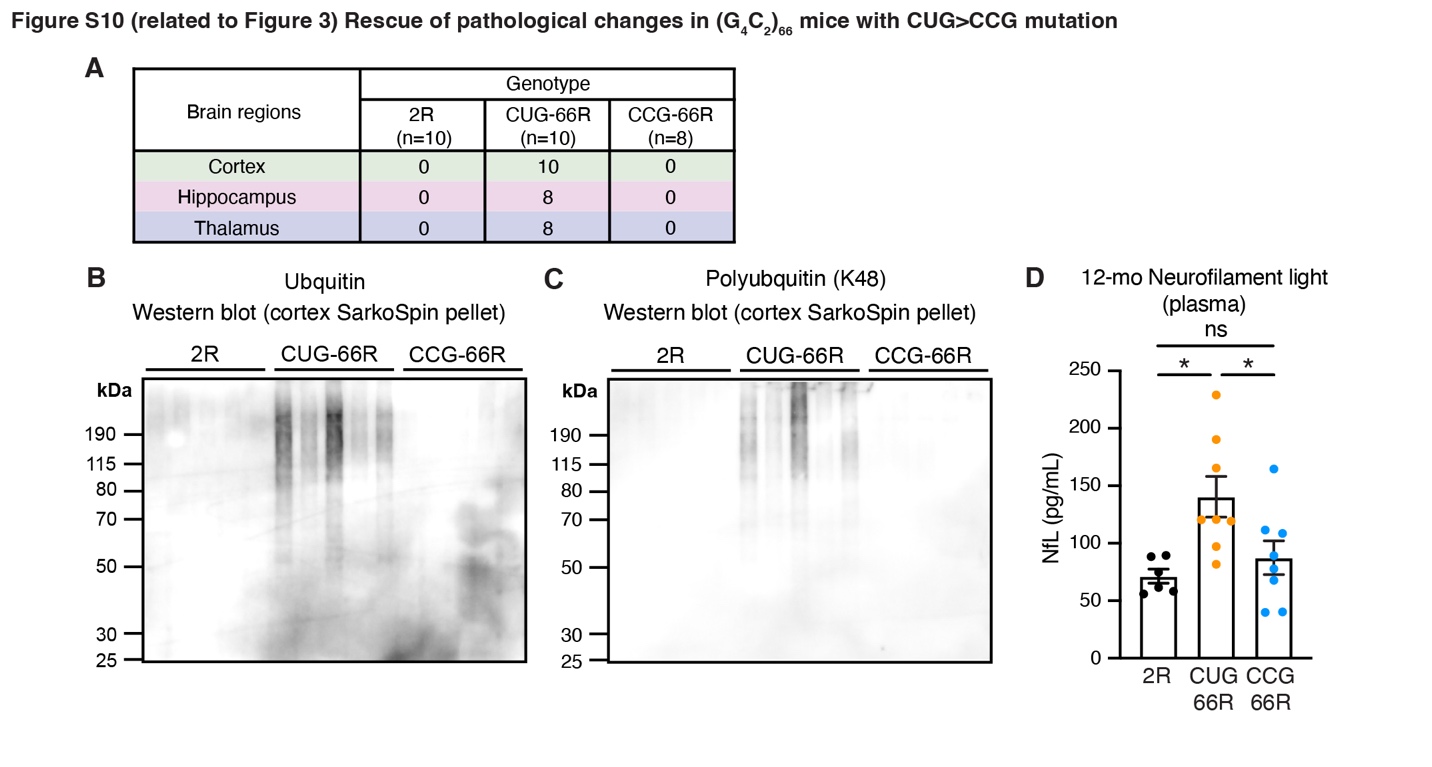


**Fig. S10 Rescue of pathological changes in (G_4_C_2_)_66_ mice with CUG>CCG mutation.** (**A**) Summary of the number of mice with p-TDP-43-positive aggregates in different brain regions by immunohistochemistry staining. (**B, C**) Immunoblot showing the level of ubiquitin (**B**) and polyubiquitin (K48) (**C**) in the SarkoSpin pellet fraction from cortex of 15-month-old mice. N=5 mice per genotype. (**D**) Neurofilament light (NfL) concentration in plasma of 12-month-old mice. N= 6-8 mice per genotype, mean ± SEM, one-way ANOVA with Tukey’s multiple comparisons test, * P<0.05.


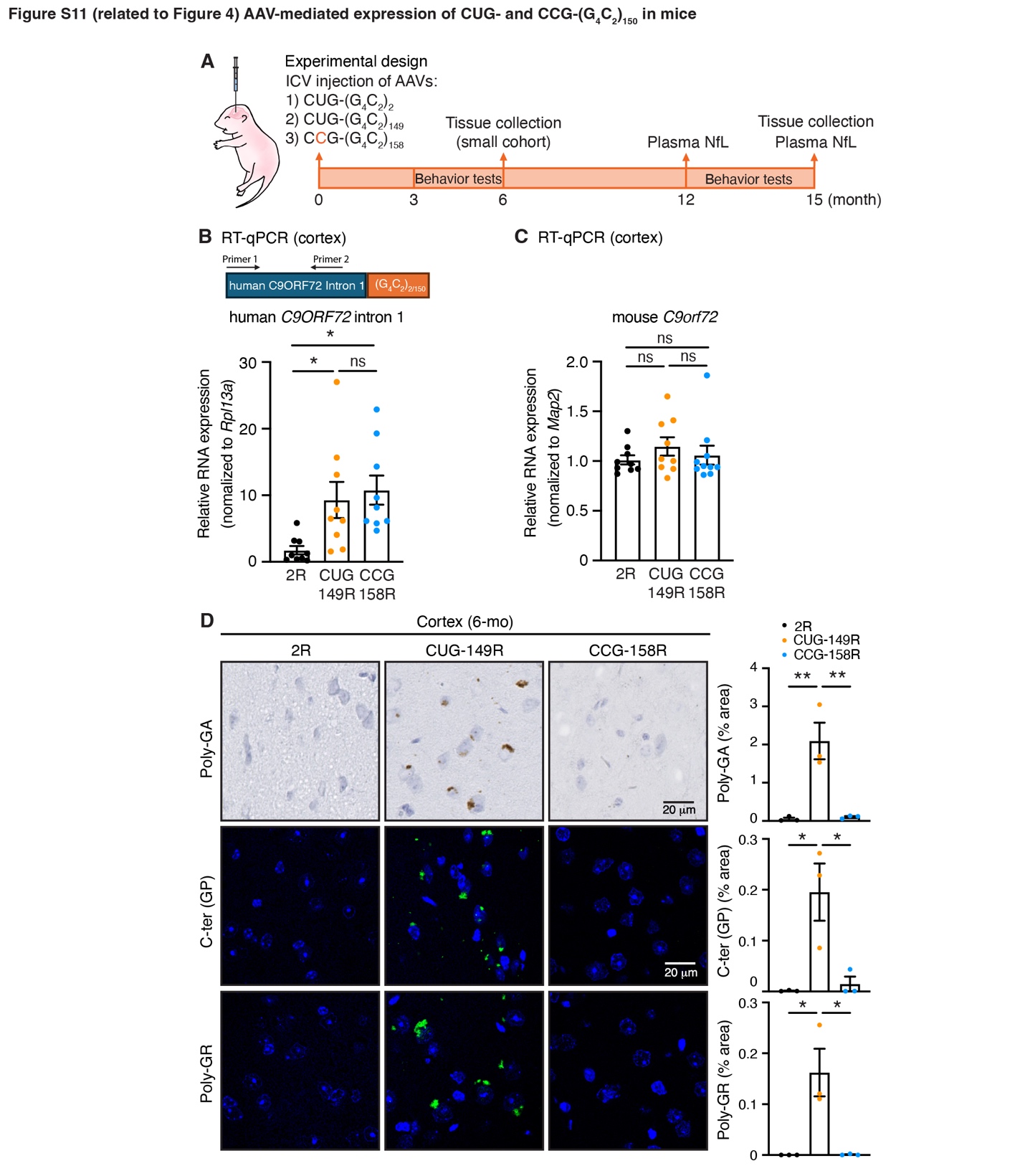
**Figure S11**

**Fig. S11. AAV-mediated expression of CUG- and CCG-(G_4_C_2_)_150_ in mice**. (**A**) Schematic of *in vivo* experiments performed to evaluate the contribution of RAN translation in *C9ORF72* mice expressing (G_4_C_2_)_149/158_ with either a CUG or mutated CCG codon upstream of the repeat (**B**) RT-qPCR determining the levels of the transgene using primers in the human *C9ORF72* intron 1b in cortex of 10-month-old AAV-injected mice. N = 9 mice per genotype, each dot represents the value from one animal. Mean ± SEM; one-way ANOVA with Tukey’s multiple comparisons test; * P<0.05. (**C**) RT-qPCR determining the levels of the mouse *C9orf72* in cortex of 18-month-old AAV-injected mice. N = 9 mice per genotype, each dot represents the value from one animal. Mean ± SEM; one-way ANOVA with Tukey’s multiple comparisons test. (**D**) Immunostaining of cortex from 6-month-old mice with antibodies against poly-GA, C-terminal peptide in frame with poly-GP (C-ter GP), and poly-GR. The percentages of area covered by DPR-positive pixel were quantified. N = 3 mice per genotype, each dot represents the average value from two brain sections from the same mouse. Mean ± SEM; one-way ANOVA with Tukey’s multiple comparisons test; * P<0.05, ** P<0.01.

**
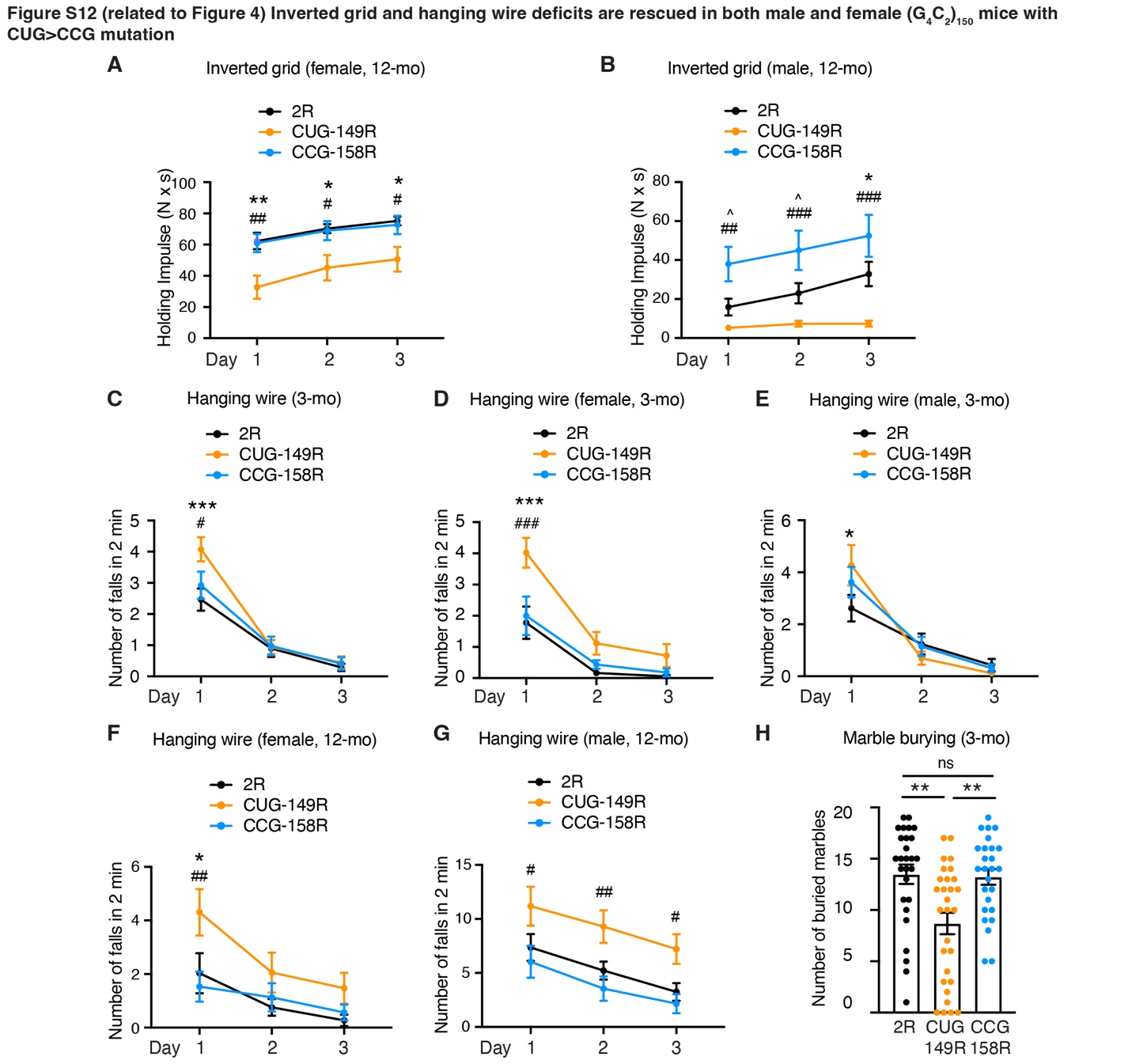
Figure S12**

**Fig. S12. Inverted grid and hanging wire deficits are rescued in both male and female (G_4_C_2_)_150_ mice with CUG>CCG mutation.** (**A, B**) Inverted grid test performed in 12-month-old female (**A**) and male (**B**) mice. Holding impulse = body weight (g) x 0.00980665 (N/g) x hanging time (s). N ≥ 10 mice per genotype/sex. (**C-G**) Hanging wire test determined the number of falls within 2 min in 3-month-old (**C to E**) and 12-month-old (**F, G**) female and male mice. n≥ 10 mice per genotype/sex, mean ± SEM, two-way ANOVA with Tukey’s multiple comparisons test, *: differences between 2R and CUG-149R; #: differences between CUG-149R and CCG-158R; ^: differences between 2R and CCG-158R. * P<0.05, ** P<0.01, *** P<0.001. (**H**) Marble burying assay was performed in 3-month-old mice. N ≥ 26 mice per genotype, mean ± SEM, one-way ANOVA with Tukey’s multiple comparisons test. ** P<0.01.

**
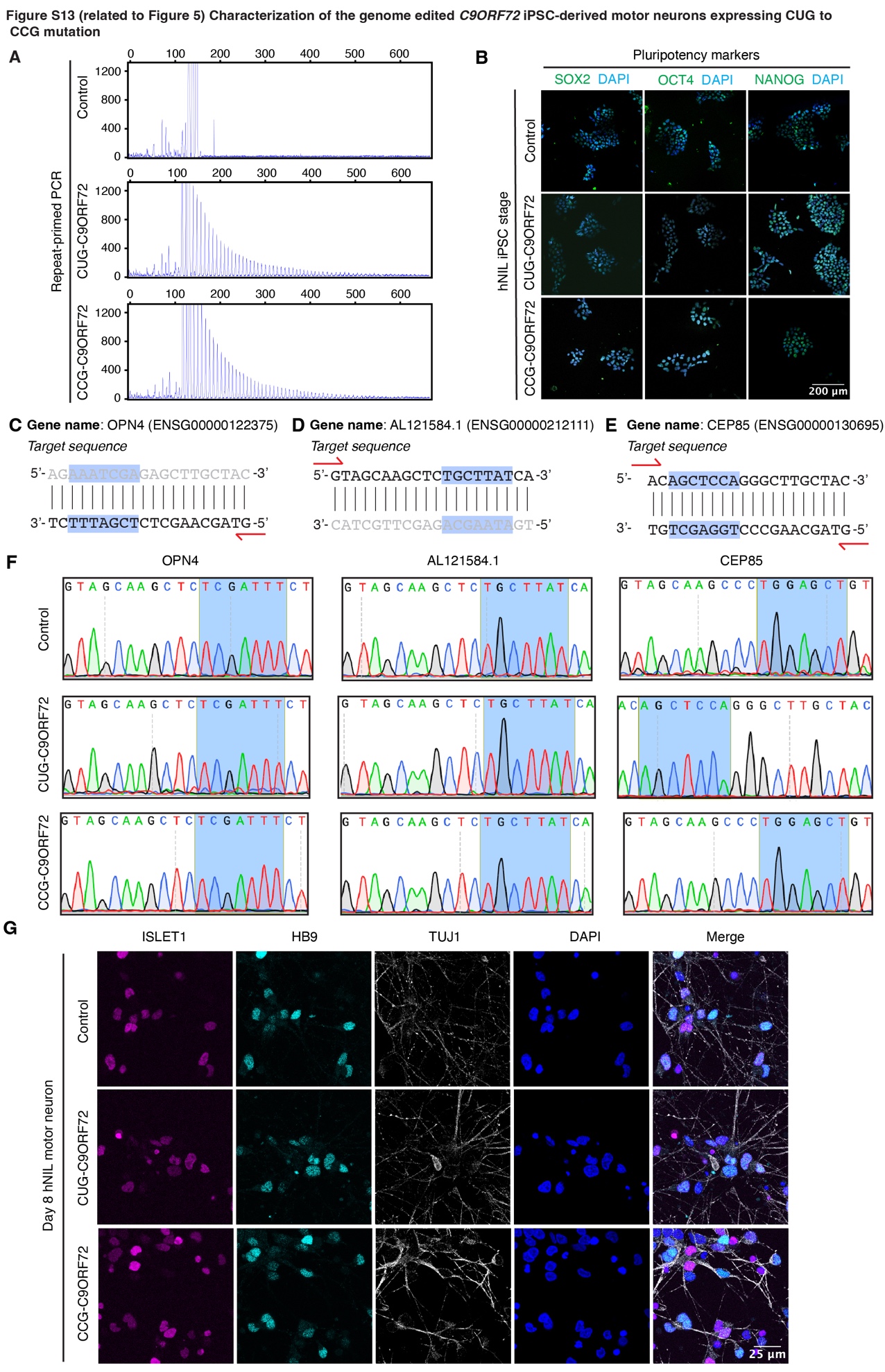
Figure S13**

**Fig. S13 Characterization of the genome edited *C9ORF72* iPSC-derived motor neurons expressing CUG to CCG mutation.** (**A**) Electropherograms showing the PCR products of repeat-primed PCR reactions from control, *C9ORF72*, and CUG>CCG edited *C9ORF72* iPSCs. (**B**) Immunostaining showing the expression of pluripotency-associated markers SOX2, OCT4, and NANOG, in control, *C9ORF72*, and CUG>CCG edited *C9ORF72* iPSCs. (**C-F**) Top potential off target sites of base editing predicted by CCTop were analyzed by Sanger sequencing. (**C-E**) Schematic showing the optimal protospacer of the top potential off-target loci in the *OPN4*, *AL121584* and *CEP85* genes. Red arrows indicate the sequencing primers used for Sanger sequencing. The sequenced strands are indicated in black, while the non-sequenced strands are in grey. The potential editing windows are labeled with a blue background. (**F**) Sanger sequencing of the potential editing window for each gene showing that no off-target mutation was induced at these loci in *C9ORF72* iPSCs that underwent the base editing process. (**G**) Immunostaining with antibodies against the motor neuronal markers Islet1 and HB9, and pan neuronal marker TUJ1 in hNIL motor neurons after eight days of differentiation.

**
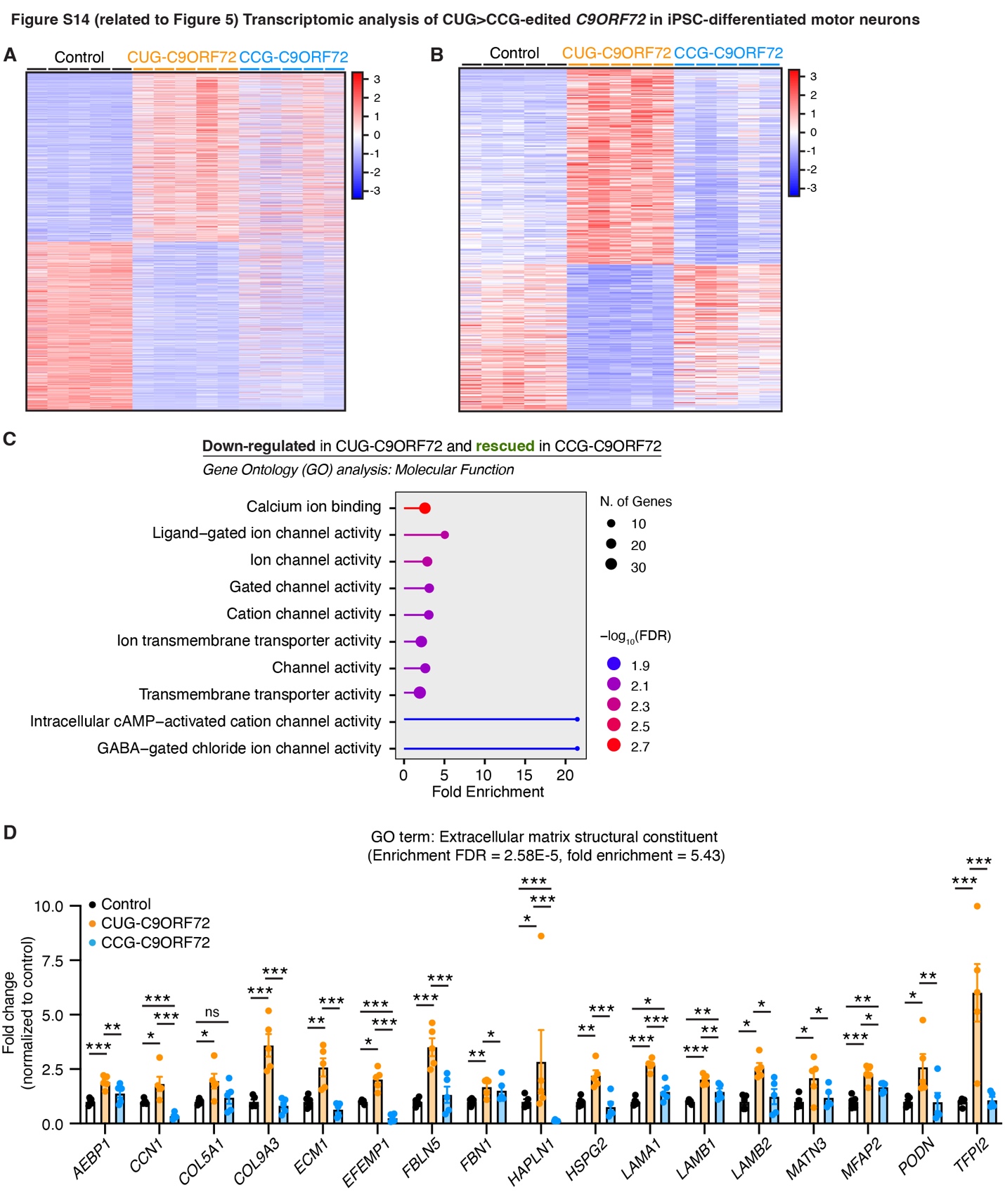
Figure S14**

**Fig. S14 Transcriptomic analysis of CUG>CCG-edited *C9ORF72* in iPSC-differentiated motor neurons.** (**A**) Heatmap showing gene expression differences among healthy control, CUG-*C9ORF72*, and CCG-*C9ORF72* motor neurons. Genes are ordered by log₂ fold change between healthy control and CUG-*C9ORF72* groups. Expression levels are displayed as Z-score–transformed normalized counts, where 0 (white) represents the mean expression across all samples, positive values (shades of red) indicate above-average expression, and negative values (shades of blue) indicate below-average expression. The color intensity reflects the number of standard deviations from the mean (e.g., ±3 corresponds to expression levels 3 standard deviations above or below the mean). **(B)** Heatmap showing genes that were differentially expressed between the healthy control and CUG-*C9ORF72* neurons and whose expression was rescued in CCG-*C9ORF72* neurons**.** (**C**) Gene Ontology (GO) Molecular Function terms enriched in genes that are down-regulated in CUG-*C9ORF72* and rescued in CCG-*C9ORF72* motor neurons (ranked by False Discovery Rate, FDR < 0.05). The length of the lines represents the fold enrichment of each term, the size of the dots correlates with the number of genes in each term, the color represents their FDR values. (**D**) Normalized TPM values of genes from the significantly changed GO term. Mean ± SEM, n = 5 biological replicates/genotype from 3 independent differentiation experiments, P values were derived from DESeq2 analysis, * P<0.05, ** P<0.01, *** P<0.001.

**
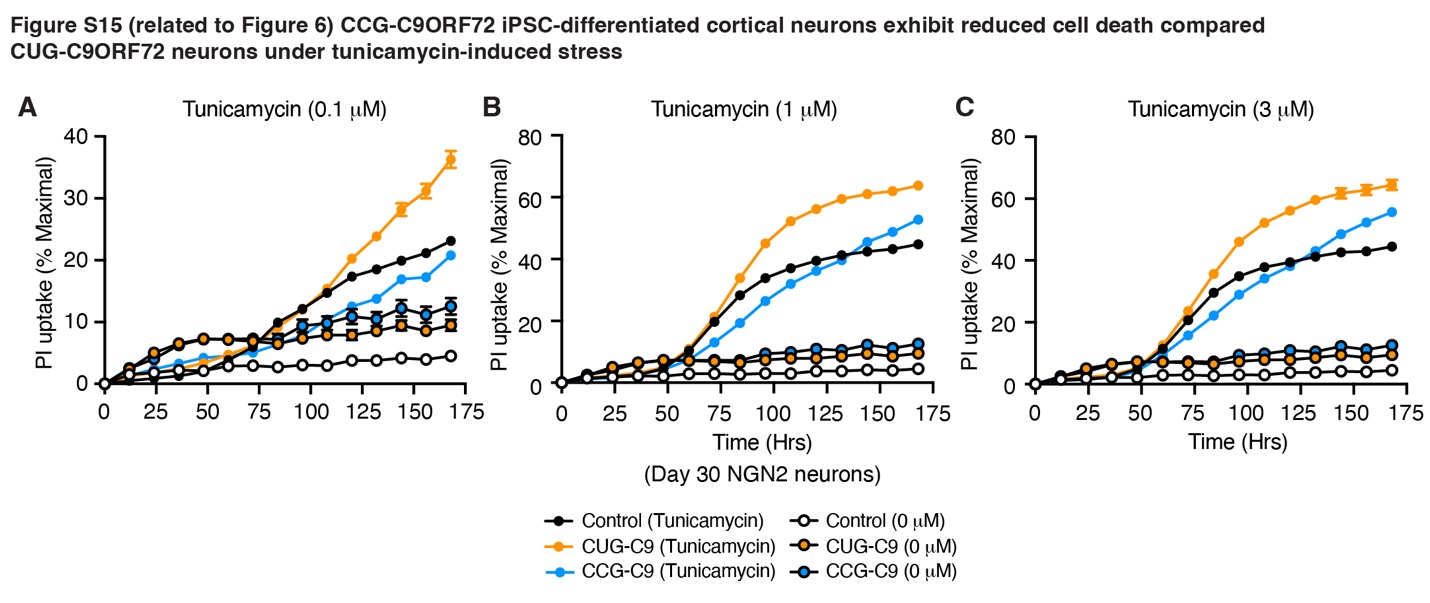
Figure S15**

**Fig. S15 CCG-*C9ORF72* iPSC-differentiated cortical neurons exhibit reduced cell death compared CUG-*C9ORF72* neurons under tunicamycin-induced stress**. (**A-C**) NGN2 neurons were incubated in propidium iodide (PI)-containing medium and treated with 0.1 μM (**A**), 1 μM (**B**), and 3 μM (**C**) tunicamycin. Images were acquired every 12 hours over 7 days (N=16 wells/genotype; three images per well per time point); mean ± SEM.

**Table S1: RNA-seq analysis of iPSC-derived motor neurons**

Transcripts Per Million (TPM) values, normalized counts, differential gene expression analysis between different groups, and the set of genes rescued in CCG-*C9ORF72* neurons (log₂FC > 0.5; adjusted *P* < 0.05) are provided in different tabs.

**Table S2: Gene Ontology (GO) analysis of genes rescued in CCG-*C9ORF72* compared to isogenic CUG-*C9ORF72* iPSC-derived neurons**

Molecular function, biological process and cellular component GO terms enriched among genes dysregulated in CUG-C9ORF72 versus control neurons and rescued in the CCG-C9ORF2 line.

**Table S3: List of antibodies**

Source, catalog number, and working dilution for all antibodies used in this study.

**Table S4: List of oligonucleotides**

Sequences of primers, probe, and sgRNA used in this study.
